# Reusable modular architecture enables flexible cognitive operations

**DOI:** 10.21203/rs.3.rs-7420993/v1

**Published:** 2025-09-19

**Authors:** Yuma Osako, Timothy J. Buschman, Mriganka Sur

**Affiliations:** 1 Picower Institute for Learning and Memory, Massachusetts Institute of Technology, Cambridge, MA 02139, USA; 2 Laboratory for Haptic Perception and Cognitive Physiology, RIKEN Center for Brain Science, Saitama 351-0198, Japan; 3 Princeton Neuroscience Institute, Princeton University, Washington Rd, Princeton, NJ 08540, USA; 4 Department of Psychology, Princeton University, Washington Rd, Princeton, NJ 08540, USA; 5 Department of Brain and Cognitive Sciences, Massachusetts Institute of Technology, Cambridge, MA 02139, USA

## Abstract

Complex behaviors are thought to be built by combining simpler cognitive components. Computational modeling has shown that artificial neural networks can perform a variety of tasks by flexibly combining small functional modules of neurons, each specialized for a specific computation, to construct a complex task. However, empirical evidence for such reusable modular networks in the brain has been lacking. Here, we show that mice performing a delayed match-to-sample with delayed report (DMS-dr) task reuse subspaces of neural activity that were specialized for stimulus processing and memory maintenance. These subspaces were reused during the task to represent new stimulus inputs and different types of memories, respectively. Clustering analyses showed each subspace was supported by a functionally distinct cluster of neurons in medial prefrontal cortex (mPFC) and posterior parietal cortex (PPC). Recurrent neural networks trained to capture the observed neural dynamics demonstrated that silencing specific clusters disrupted specific computations, highlighting the modular and reusable organization of these networks. By bridging theoretical predictions with empirical evidence, our findings suggest that the brain can flexibly reuse computational components to perform a complex cognitive task.

## Introduction

Real-world behavior in complex environments requires the brain to flexibly coordinate multiple cognitive operations, such as processing sensory inputs, maintaining information in memory, and generating decisions^1–3^. Theoretical models have posited that each of these operations can be performed in independent modules and then reused in different contexts or at different times within the same task^4,5^. This reusability and modularity is thought to allow existing resources to be utilized and combined in different ways, allowing the brain to perform a wide array of cognitive operations and flexibly adapt to new tasks^4–7^. Importantly, reusing neural computations reduces the need to represent all possible combinations of information processing for every task, thereby conserving neuronal resources and achieving the brain’s capacity for generalization. However, despite its theoretical significance, the neuronal circuit mechanisms underlying such reuse of cognitive operations remain largely unknown.

**Fig. 1 |:**
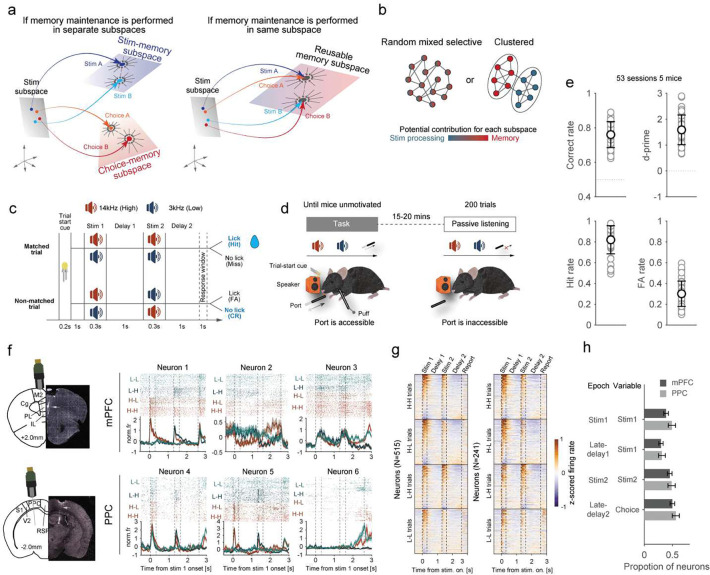
Behavioral performance and neuronal recordings during the DMS-dr task. **(a)** Opposing hypotheses for the neuronal representation of memory content in activity space. One hypothesis (left) posits that distinct information, such as stimuli or choice, is encoded in the different subspaces in neural activity space. Alternatively (right), a shared representational subspace might be utilized across different kinds of information. **(b)** Opposing hypotheses for the functional organization within neuronal populations. One hypothesis (left) suggests that neurons exhibit random mixed selectivity for information processes (e.g., stimulus processing, memory maintenance), with no clear functional clustering. The alternative hypothesis (right) proposes that neuronal selectivity is organized into functionally distinct clusters, where separate subpopulations of neurons are specialized for specific processes or information types. **(c)** Timeline and trial types for the DMS-dr task. Licking was assessed during a 1s response window. **(d)** Experimental setup for the active (left) and passive (right) blocks. **(e)** Correct rate, d-prime, hit rate, and false alarm (FA) rate for all mice (53 sessions, 5 mice). **(f)** Electrophysiological recordings using a high-density probe and neuronal activity of example neurons. Top: spike raster. Bottom: peri-stimulus time histogram (PSTH), with colors representing four different trial types. **(g)** Proportion of neurons encoding stimulus 1, stimulus 2 and choice across different task epochs for mPFC and PPC. **(h)** Neuronal responses for all neurons in mPFC and PPC to the different trials. Neurons are sorted by response magnitude during the presentation of stimulus 1. Error bar represents binomial confidence intervals.

Previous studies in humans^8–11^, non-human primates^12–15^, and rodents^16,17^ have found that task-relevant information is encoded in an abstract, compressed representation that can be reused across contexts. However, these findings primarily focused on the reusability of information representations with respect to a specific type of information, e.g., reusing the representation of a sensory stimulus for different tasks^9,11,12,14,17^. It remains unknown whether the same neural circuit can be repurposed to perform the same computation on different types of information at different times, e.g., maintaining a memory ^18–21^ of a sensory stimulus and then maintaining a memory of an upcoming response (Fig.1a). Addressing this question is critical for understanding the hierarchical level at which the brain reuses neural circuits - whether reuse occurs at the level of representations or computations - thereby providing essential insight into the hierarchy of reusability in neural circuits. Furthermore, while modeling studies suggest that reusable computations are implemented by distinct subpopulations of neurons^4,5^, it is unknown whether this is true in the brain (Fig.1b). Evidence from previous experimental studies has been divided, with evidence that neurons exhibit random mixed selectivity that contributes to multiple task variables^22,23^ and evidence that neurons form distinct clusters, each dedicated to a specific computational function ^24,25^.

To address these questions, we developed a delayed match-to-sample with delayed report (DMS-dr) task in mice. This task requires mice to process stimuli and hold task-relevant information during two stimulus presentations and two delay periods, respectively. We performed electrophysiological recordings to examine neural dynamics in the medial prefrontal cortex (mPFC) and posterior parietal cortex (PPC) during the task. Our findings revealed that different subspaces of neural activity were associated with sensory processing and memory maintenance. Importantly, these subspaces generalized across the content being represented – the same memory subspace was used to maintain stimulus information and motor response information. Furthermore, these two distinct computations were supported by distinct functional clusters of neurons. When these clusters were inhibited in recurrent neuronal networks (RNNs) trained to reproduce the mouse’s neural activity, the network showed specific deficits in either sensory or memory processes, highlighting the modular architecture that supports flexible cognitive behavior.

## Results

### A task that requires mice to process stimuli and maintain memories twice

We trained mice to perform a delayed matched to sample, delayed response (DMS-dr) task (Fig.1c–d). In this task, water-deprived, head-fixed mice were trained to compare two auditory stimuli to determine whether they were the same or different. The stimuli were pure-tone 0.3s auditory stimuli with either a high (H, 14kHz) or low (L, 3kHz) frequency. The first and second stimuli were separated by a 1s memory delay. After the second stimulus, there was another 1s delay before the animal reported whether the two stimuli matched, by licking a reward spout, or did not match, by refraining from licking. Correct licks (hits) were rewarded with water, while incorrect licks to non-match stimuli (false alarm) resulted in an air-puff and a 7s-timeout (Fig.1c). Trials in which the mice refrained from licking after matched stimuli (miss) or after non-match stimuli (correct reject) were not reinforced.

Mice performed the task well, with an accuracy of 76±7%, a discriminability index (d’) of 1.6±0.6, a hit rate of 0.82±0.13, and a false alarm rate of 0.30±0.12 (Fig.1e, mean ± SD). Behavioral performance was above chance for all stimulus pairs (Supplementary Fig.1a) and slightly improved after correct trials compared to error trials (Supplementary Fig.1b). To further assess the factors influencing behavioral choice, we fitted a logistic regression model to predict the animal’s response based on the current stimuli and previous trial information (e.g., correctness, stimulus, choice, false alarm, and hit). The model performance was cross-validated (Supplementary Fig.1c, Methods) and showed that mice made decisions primarily based on the current stimuli, with little influence of previous trial information (Supplementary Fig.1d). Together, these results demonstrate that the mice processed the stimuli and maintained this information in working memory to guide behavior.

To compare neural representations across different tasks, mice also performed a passive listening task after the DMS-dr task (Fig.1d). This task was identical to the active task except that the lick spout remained retracted so that the mice did not respond (Supplemental Video 1).

Given the established role of the mPFC and PPC in stimulus processing^26^ and memory maintenance^27,28^, we first tested whether these regions were causally required for the DMS-dr task. We optogenetically inactivated the CaMKII^+^ neuronal activity of the mPFC or PPC during six different epochs using a soma-targeted anion channelrhodopsin (stGtACR2) (Supplementary Fig.1e–f). Inactivation of these areas led to (1) a general decreased correct rate and d-prime across nearly all inhibition epochs, except during the early-delay 1 epoch following PPC inhibition; (2) a decreased hit rate during the stimulus and delay 1 epochs, but an increased hit rate during the delay 2 epoch; and (3) a paradoxical increased false alarm rate across all epochs, except during early delay 1 in both mPFC and PPC, and during late delay 2 in PPC (Supplementary Fig.1g). These results suggest that mPFC and PPC neurons are engaged and are necessary across various epochs of the task.

### Neuronal subspaces are shared across different epochs

**Fig. 2 |:**
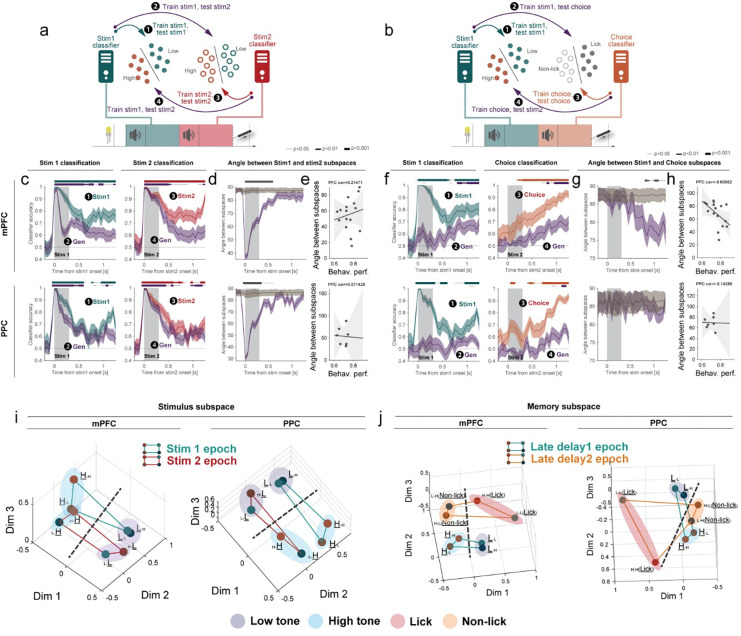
Neural subspaces are shared during the task **(a-b)** Schematic of classifiers used to quantify stimulus and choice information. Stimulus 1, stimulus 2, and choice information are evaluated during [−0.2s 1.3s] from stimulus 1 or 2 onset. Cross-conditional classification was performed by decoding stimulus 1 (or 2) using a classifier trained on stimulus 2 (or 1) (**a,** ❷ and ❹) or by decoding stimulus 1 (or choice) using a classifier trained on choice (or stimulus 1) (**b,** ❷ and ❹). **(c)**. Time course of classifier accuracy for stimulus 1 and 2 information in each region. Horizontal bars (top of each plot) indicate above-chance classification (P < 0.05, 0.01, and 0.001 for thin, medium, and thick lines, respectively; bootstrap test) **(d)** Time course of the angle between stimulus1 and 2 subspaces for each region. **(e)** Angle between stimulus 1 and 2 subspaces as a function of behavioral performance. Each dot represents a single session (N=16 or 7 sessions for mPFC and PPC, respectively). Spearman’s rank-order correlation coefficient was used, and *p*-values were computed by permutation test. **(f)** Time course of classifier accuracy for stimulus 1 and choice information in each region. **(g)** Time course of the angle between stimulus1 and choice subspaces for each region. **(h)** Angle between stimulus 1 and choice subspaces as a function of behavioral performance. Each dot represents a single session (N=16 or 7 sessions for mPFC and PPC, respectively). **(i-j)**. Representations of different trials during the stimulus epoch (0–0.5s and 1.3–1.8s from stimulus 1 onset) (**i**) and the late-delay epoch (0.8–1.3s and 2.1–2.6s from stimulus 1 onset) (**j**) for each region. Lines and shading represent mean ± std for **c**, **d**, **f**, and **g**. For **e** and **h**, lines are fitted with least-squares linear regression. Shading denotes the 95% confidence interval of the regression.

To understand how the mPFC and PPC were engaged in the task, we performed dense silicon electrode recordings (Neuropixels probes^29^) from mPFC (515 neurons, n=6 mice) and PPC (241 neurons, n=4 mice) simultaneously (Fig. 1d, left, supplemental table 1). Neurons in both regions represented task-relevant variables, including the identity of the first and second stimulus and the animal’s behavioral response. This information was represented in single neurons (Figs.1f–h and Supplementary Fig.2) and in the neural population (Supplementary Fig.3). To quantify information about these task variables without sampling bias, we trained logistic regression classifiers using all neurons pooled across experiments (“pseudo-population^30^”) to decode stimulus (high/low-tone) and choice (lick/non-lick) (Fig.2, a and b, ❶ and ❸). Classifiers trained on mPFC and PPC neural activity accurately decoded both stimulus and choice information (Fig.2c and f, ❶ and ❸, p<0.05 bootstrap test, and Supplementary Fig.4a; performance was measured on cross-validation trials). As expected, classifiers for stimulus 2 and choice performed at chance level prior to the presentation of the stimulus 2, indicating that this information was not represented by the neural population until after the stimulus 2 (Supplementary Fig.4A). We also applied cross-temporal classification to assess the stability of the population code over time (Supplementary Fig.4b; Methods). This analysis revealed that the population code dynamically shifted immediately following the stimulus presentation (0–0.5s from stimulus 1) into the memory delay (0.8–1.3s from stimulus 1) (Supplementary Fig.4c), suggesting that mice utilize different population subspaces during these epochs ^31,32^.

These results suggest that there are dedicated subspaces of neural activity that perform specific cognitive computations – one for processing sensory stimuli and one for maintaining information in memory. If this is true, then the same population representation could be re-used, either at different times during the task or to encode different types of information. To test this hypothesis, we trained logistic regression classifiers to decode stimulus identity (low, L, vs high, H, tones) or choice (lick vs non-lick; i.e., (L-L + H-H) vs (L-H + H-L) trials) from the neuronal population activity and quantified how well these classifiers generalized across conditions and epochs^9,12,13^ (Fig.2 a–b, purple arrow, ❷ and ❹). In both mPFC and PPC, classifiers trained to decode the identity of the stimulus during one stimulus epoch generalized this discriminability to the other stimulus epoch ^14^ (Fig.2c, purple ❷ and ❹, p<0.05, bootstrap test, Supplementary Fig.5, a and c). In other words, a classifier trained on stimulus 1 decoded stimulus 2, and vice versa (Fig.2c, stim 2 classifications, purple). To evaluate if the stimulus subspaces overlapped, we quantified the angle between stimulus 1 and 2 period classifiers at each timepoint and found the subspaces were significantly more aligned than expected by chance but gradually became orthogonal during the delay period (Fig.2d). The angle between sensory 1 and sensory 2 subspaces was not correlated with the behavioral performance of animals (Fig.2e). We additionally found that the shared sensory subspace was also engaged during the passive task (Supplementary Fig.5f, p<0.05, bootstrap test), indicating that it generalizes across tasks. Together, as expected, these cross-condition classification analyses suggest that a common neural circuit is used to represent the identity of a stimulus, regardless of the time during the task and even the passive session.

In the DMS-dr task, mice were required to hold two distinct types of information during the first and second memory delays: during the first memory delay, they had to remember the recent sensory stimulus, and during the second memory delay, they remembered the upcoming behavioral response (Fig.1a). Despite these being different types of information, we were curious as to whether the same subspace was used to maintain both memory representations. In mPFC, classifiers trained to decode stimulus identity during delay 1 significantly decoded choice during delay 2, and vice versa (Fig.2f, purple ❷, p<0.05, bootstrap test, Supplementary Fig.5a). In other words, a classifier trained to decode the identity of stimulus 1 during the first memory delay was able to decode the animal’s upcoming choice during the second memory delay, and vice versa (Fig.2f, choice classifications, purple ❹). These shared subspaces were not attributable to a selectivity bias towards the tone (Supplementary Fig.5b) or due to the licking action itself (Supplementary Fig.5e) and the same effect was seen in single-sessions (Fig.S5C). Note that the identity of the first stimulus and choice were not correlated due to balanced trial numbers across conditions (see Methods). In contrast to the shared stimulus subspace (Fig.2d), the angle between the subspace representing the memory of the first stimulus and the memory of the upcoming choice decreased throughout the delay period in the mPFC (Fig.2g). Similar results were also found using a generalized linear model (Supplementary Fig.6). Together, these results suggest that, in mPFC, there was a dedicated subspace of neural activity that maintained memories, regardless of the exact type of information being maintained (e.g., both the memory of the first stimulus and the memory of the upcoming choice). The shared subspace was stronger in mPFC compared to PPC (Fig.2g).

The engagement of this shared subspace was related to the task. In the mPFC, but not in PPC, lower angles between classifiers, and thus greater subspace overlap, was correlated with better behavioral performance (Fig.2h and Supplementary Fig.5c, bottom). This correlation emerged during the late-delay period (Supplementary Fig.5d). Furthermore, the same subspace was not observed during the passive task; the classifier trained to decode choice (in delay 2) during the DMS-dr task session did not decode the memory of the stimulus in the passive task (Supplementary Fig.5g). These findings suggest that the shared subspace for memory maintenance is engaged in a task-specific manner.

Finally, we were interested in whether the shared subspaces preserved the geometry of representations within the neural population. So, we investigated how the neural population represents different trial types by projecting the neural representation of four different trial types (stimulus 1 and 2 pair: Low-Low, Low-High, High-Low, High-High) in the mPFC and PPC into a reduced dimensional space specific to stimulus and late-delay epochs (Method). Projection in the stimulus subspace revealed relatively parallel and aligned coding direction of stimuli (high or low tones) (Fig.2i). This geometric structure permits a single decision boundary (Fig.2i, dotted line) to decode stimulus identity in both stimulus 1 and 2 epochs, suggesting that this boundary is reusable across these stimulus presentations as expected ^11–13^. Projection in the memory maintenance subspace also revealed that a single decision boundary can be used to differentiate stimulus identity and choice (lick or non-lick). However, this boundary was more obvious in mPFC compared to PPC. These geometries emerged only at the corresponding times during the trials (Supplementary Fig.7, Task, stimulus subspace: 0–0.5s and 1.3–1.8s, memory maintenance subspace: 0.8–1.3s and 2.1–2.6s) and only the stimulus subspace exhibited the same geometry in the passive task (Supplementary Fig.7, Passive).

Altogether, these results suggest that, as expected, the neural subspace for stimulus representation is dynamically reused at appropriate times in both the mPFC and PPC. In contrast, the subspace for memory maintenance exhibits reusability especially in the mPFC. Notably, memory maintenance subspaces are reused even when the information they encode is different, suggesting that this subspace serves a specific cognitive computation rather than merely reflecting the physical similarity of stimuli. The importance of this subspace is highlighted by the fact that the animals’ behavioral performance was greatest when the memory maintenance subspace in the mPFC was more shared.

### Single neuron clusters represent distinct neuronal dynamics

**Fig. 3 |:**
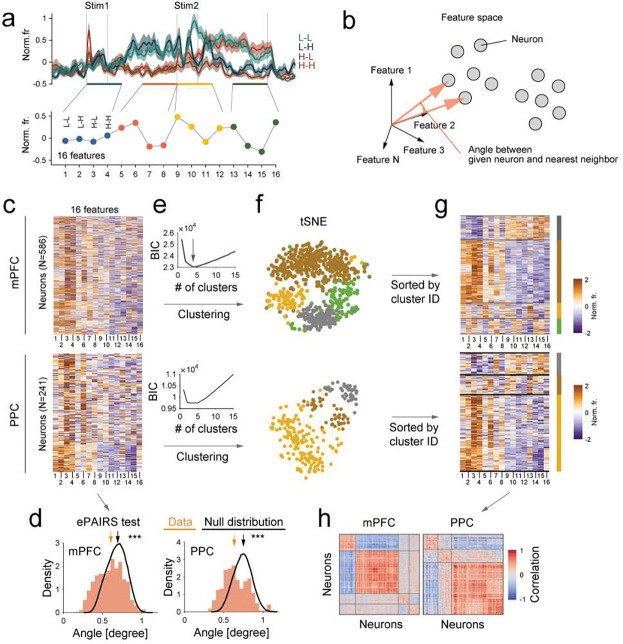
mPFC and PPC neurons form discrete clusters **(a)** Top: neural response of an example neuron across all four trial types. Bottom: response vector calculated as the mean response during each epoch (0–0.5s, 0.8–1.3s, 1.3–1.8s, and 2.1–2.6s from stimulus 1 onset). **(b)** A 16-dimensional vector represents each neuron’s activity to each feature. For neuronal populations with random mixed selectivity, vectors are uniformly distributed around the origin, which can be quantified by angles between nearest neighbors (ePAIRS). **(c)** Unsorted response vectors of all neurons in the mPFC. **(d)** Distribution of angles between each point and its nearest neighbor in the feature space compared to that of a matching multivariate Gaussian (null distribution, black line) for each brain region. *p*<0.001, ePAIRS test (two-sided, see Methods). **(e)** BIC scores used to determine the optimal number of clusters. **(f)** Visualization of the response vectors using t-distributed stochastic neighbor embedding (t-SNE). Each dot represents a single neuron, colored according to its cluster membership. **(g)** Response vectors sorted by their respective cluster membership. **(h)** Sorted correlation matrices revealing strong within-cluster similarity.

Thus far, we have shown that subspaces are shared at appropriate times during the task, supporting the concept of reusable cognitive operations. These operations could be interpreted either in terms of collective dynamics from all neurons (Fig.1b, left, Random mixed selective)^22,23,33^ or as subpopulations of distinct neurons^24,25^ (Fig.1b, right, Clustered). To distinguish between these hypotheses, we employed two approaches. First, we examined the response profile of individual neurons during the task. A response vector was created for each neuron by concatenating its responses across the four trial types (L-L, L-H, H-L, H-H) during the stimulus and late-delay epochs for both the first and second stimuli (Fig.3a, 4 trial types × 4 epochs = 16 dimensions). This response vector from all neurons allowed us to examine the distribution of neurons within the feature space. A neuronal population that is randomly mixed within the representational space would have uniformly distributed response vectors around the origin. To test this, we applied the ePAIRS test (Fig.3b, Methods)^22,24^ to compare the empirical distribution with a randomized shuffle corresponding to a multivariate Gaussian (Fig.3c–d). Both mPFC and PPC populations had non-uniform distributions, significantly different from the artificial uniform distribution (Fig.3d, p<0.001, ePAIRS test, Methods). This indicates that individual neurons in these regions do not uniformly occupy the space of all possible representations but instead have functional specializations. This non-random distribution was not observed when using neuronal activity during pre-stimulus period (Supplementary Fig.8a), suggesting functional specialization is associated with information processing.

Next, we tested whether neurons clustered into functional modules. Fitting a Gaussian mixture model (GMM) to the response vectors (Fig.3c, e–g, Methods) revealed four functional clusters in mPFC and three functional clusters in PPC (Fig.3e; chosen based on minimizing Bayesian information criterion, BIC, scores). For visualization, we used tSNE ^34^ to project neural responses into a two-dimensional space (Fig.3f). Consistent with the observed clustering, all clusters showed strong correlation of activity within the cluster (Fig.3h) and significantly greater co-fluctuation among neurons within the same cluster compared to across clusters (except mPFC-cluster 1 and PPC-cluster 2, Supplementary Fig.8c–f). The functional clusters were not distinguished by waveform of neurons or anatomical locations within mPFC and PPC (Supplementary Fig.8g–i), suggesting that functional specialization arises from network dynamics rather than fixed anatomical segregation.

**Fig. 4 |:**
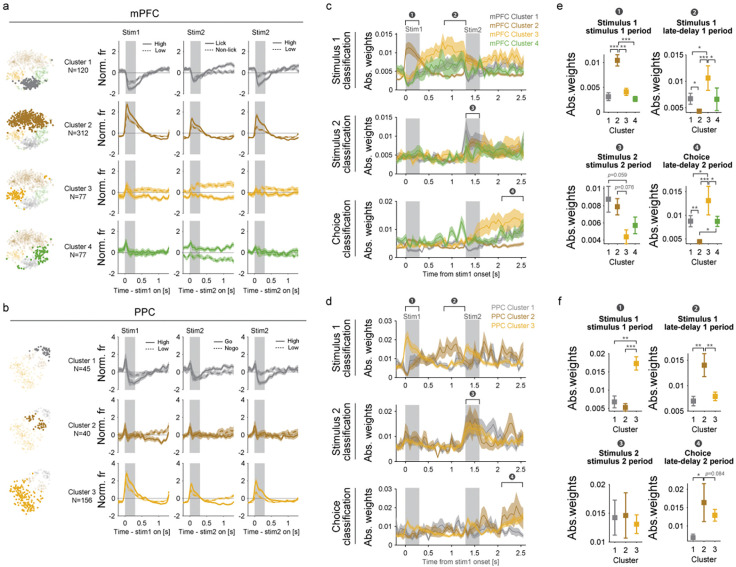
Discrete clusters dominantly contributed to subspaces at different timing **(a-b)** Average responses of neurons within each cluster. **(c-d)** Time course of absolute weights for the stimulus 1, stimulus 2, and choice classifiers. **(e-f)** Average absolute weights during the epochs indicated in **c** and **d**. ***p<0.001, **p<0.01, *p<0.05 One-way ANOVA followed by post-hoc LSD test for multiple comparisons. All plots are shown as mean ± sem.

Each cluster represented distinct patterns of neural representation (Fig.4a–b; confirmed with a linear classifier, see Supplementary Fig.8b and Methods for details). mPFC clusters 1 and 2 distinguished between high and low tones during stimulus 1 and 2 (Fig.4a, mPFC clusters 1 and 2), while neurons in mPFC cluster 3 differentiated high/low tones and lick/non-lick behavior during delays (Fig.4a, mPFC cluster 3). mPFC cluster 1 also distinguished between lick and non-lick behavior during the second delay, and mPFC cluster 4 might represent behavioral bias (Fig.4a, cluster 4). In the mPFC, we observed clusters that were selectively engaged in distinct phases of the task, such as those dedicated to processing sensory information during the stimulus periods (Fig.4a, mPFC cluster 2) or maintaining information during the delays (Fig.4a, mPFC cluster 3). In contrast, such specialized patterns of activity were less apparent in the PPC. For example, PPC clusters 1 and 3 represented sensory information during the stimulus periods but also encoded lick/non-lick behavior during the second delay, suggesting a multiplexed coding scheme in PPC ^22,35^.

Given these responses, we hereafter refer to mPFC cluster 2 and PPC cluster 3 as putative stimulus clusters and mPFC cluster 3 as the putative memory maintenance cluster. To further investigate the contributions of each functional cluster to the stimulus and memory-maintenance subspaces, we calculated the absolute weights of each cluster for the classifiers trained above (Fig.4c–f). The putative stimulus clusters, i.e., mPFC-cluster 2 and PPC-cluster 3, contributed most strongly to the sensory classifiers trained to decode the identity of the first stimulus during the stimulus 1 epoch (Fig.4c, brown line in stimulus 1 classification for mPFC. Fig.4d yellow line in stimulus 1 classification for PPC). Moreover, the mPFC-cluster 2 also contributed to the second stimulus classifier during the stimulus 2 epoch (Fig.4c and F, brown in stimulus 2 classification). For the memory classifiers, which were trained to decode first stimulus during delay 1 or choice information during delay 2 epochs, the putative memory maintenance cluster, i.e., mPFC-cluster 3 and PPC-cluster 2, contributed significantly during both delay 1 and delay 2 (Fig.4c–f, yellow in mPFC, brown in PPC). Together, these results suggest that stimulus and memory maintenance clusters are engaged by different computations; sensory processing recruits sensory clusters during stimulus epochs while memory processing recruits memory maintenance clusters during delay epochs.

### Modular lesion effect in a RNN model of mPFC subpopulations

**Fig. 5 |:**
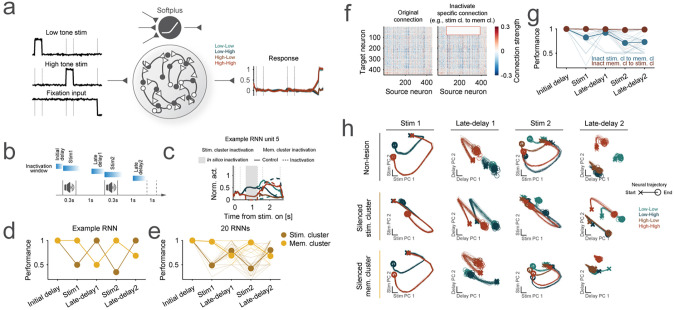
Data-constrained RNN shows modular lesion effects. **(a)** Example of a fully connected RNN architecture. The network receives stimulus and fixation inputs and projects to a fixation output unit (active when a motor response is unwarranted) and a behavioral output unit. All RNN units exhibit non-negative response. RNN containing 77 and 312 units were trained to mimic the neuronal activity of cluster 2 and 3 in the mPFC (Methods). **(b)** Schematic illustrating timing of *in-silico* inactivation of RNN clusters. **(c)** Example activity of the putative memory maintenance RNN unit during late-delay1 period, with either the putative stimulus or memory maintenance cluster inactivated. **(d)** RNN performance after silencing specific clusters during each epoch. Yellow and brown lines represent silences of the putative stimulus and memory maintenance clusters, respectively. **(e)** Summary of RNN performance after silencing clusters. Each line represents a different RNN (N=20). Stimulus epochs include stimulus 1 and 2, while delay epochs include late-delay1 and 2. Error bars indicate mean ± standard deviation **(f)** Synaptic weights between units. Inactivation of synaptic connections was simulated by setting connection weights to zero at corresponding time (right, rectangle area). **(g)**. RNN performance after silencing specific connection during each epoch. Blue lines represent silence of the connections from the putative stimulus processing cluster to memory maintenance cluster, and brown lines represent opposite direction. **(h)**. State trajectories (starting from ‘x’ and ending with ‘o’) during the performance of stimulus and late-delay periods (0–0.5s, 0.8–1.3s, 1.3–1.8s, 2.1–2.6s from stimulus onset) in silenced and full network projected into the first two PCs defined by the full network state at the stimulus and late-delay epochs, respectively.

Our data suggest there are reusable neural subspaces for stimulus processing and memory maintenance, each supported by distinct subpopulations of neurons. We next trained recurrent neural networks (RNNs) to investigate potential neural circuit mechanisms underlying the modular architecture of stimulus processing and memory maintenance observed in mPFC (Fig.5a). Our aim of this approach was to explore how distinct subpopulations within a network can give rise to flexible, epoch-dependent computations. Specifically, we sought to understand (1) how stimulus- and memory-maintenance subpopulations can interact within a network, and (2) how perturbations to these subpopulations affect task performance and network dynamics. This approach allows us to test mechanistic hypotheses about neural reusability and modularity beyond descriptive observations. The RNNs reliably reproduced the animals’ behavior and the PSTHs of mPFC neurons (Supplementary Fig.9a–b). To assess the functional role of the neuron clusters observed in Figure 4, we silenced each cluster during different epochs of the DMS-dr task (Fig.5b–c), including the initial delay, stimulus 1, late-delay 1, stimulus 2, and late-delay 2 epochs. Task performance was impaired when the mPFC-cluster 2 stimulus cluster was silenced during the stimulus epochs and when the mPFC cluster 3 memory cluster was silenced during the late-delay epochs (Fig.5d). This effect was consistent across multiple simulations (Fig.5e). Importantly, this modular lesion caused no impairment when the putative stimulus or memory maintenance cluster was not actively engaged. Examining the network state revealed that silencing the putative stimulus processing cluster during stimulus presentation (stim1 and stim2 epochs) significantly impacted state trajectories (Fig.5h, stim1 and stim2, silenced stim cluster). In contrast, silencing the putative memory maintenance cluster during these periods had minimal impact (Fig.5h, stim1 and stim2, silenced mem cluster). On the other hand, during late-delay periods, silencing the putative memory maintenance cluster altered state trajectories. Meanwhile, silencing the putative stimulus processing cluster had little effect on state trajectories although the relative positions of each trial type remained unchanged (Fig.5h, Late-delay1 and Late-delay2). Finally, we found that silencing the projection from the mPFC-cluster 2 stimulus cluster to the mPFC-cluster 3 memory maintenance cluster during stimulus epochs impaired behavioral performance (Fig. 5f to G), suggesting the putative stimulus processing cluster conveys information to the memory maintenance cluster during stimulus presentation. Altogether, these findings are consistent with a modular organization of stimulus processing and memory maintenance clusters in the network, enabling flexible computations during the task.

## Discussion

A combination of analyses of empirical neuronal data and computational simulations suggest the brain uses dedicated computational circuits for performing specific cognitive operations. Mice trained to perform a delayed match-to-sample task with a delayed response, used a ‘sensory’ subspace of neural activity in both mPFC and PPC to process incoming stimuli across multiple tasks ^9,12–15,17,36^. Moreover, we found that a ‘memory’ subspace in mPFC maintained the memory of task-relevant information. Importantly, the same memory circuit was used to maintain different types of information. In the first memory delay, the memory subspace represented the retrospective memory of the presented stimulus while, in the second memory delay, the memory subspace represented the prospective memory of the upcoming response (Fig. 2f–h, and 2j). This suggests the memory subspace engaged a specific cognitive computation – maintenance of information – that was agnostic as to the contents of the memory itself. Together, our results suggest that mPFC not only generalizes specific information representation across various contexts but can also generalize cognitive operations across different categories of information. This reuse was important for behavior: the degree to which memory maintenance subspaces were shared was significantly correlated with behavioral performance (Fig.2h and Supplementary Fig.5c, bottom). Whether these functional clusters are learned or are pre-existing remains an open question. Previous work suggests that memory maintenance neurons emerge, and their representations stabilize as animals learn the task^37^, which implies that the brain might co-opt existing modules, such as a memory maintenance module, during learning^38^.

It has been debated whether neurons are organized into clusters according to their functional roles^24,25^ or if responses are randomly distributed^22,23^. Recent work has typically found a non-modular architecture in most brain areas^39^. In contrast, we found functional clustering (see also ^24,25^). Neurons in both regions were clustered according to their response patterns during the active task (Fig.3), with specific clusters contributing predominantly to stimulus processing and memory maintenance subspaces, respectively (Fig.4). Moreover, when ablating these clusters in RNNs trained to reproduce our experimentally observed neural dynamics, we found the sensory and memory clusters impaired specific aspects of behavior and disrupted the corresponding functional dynamics of neural activity (Fig.5). Together, our results suggest that there are modules of neurons performing cognitive operations and that these modules can be flexibly reused across time and tasks.

The discrepancy between our results and previous work might stem from differences in the behavioral tasks used. Studies reporting non-modular architectures often utilize tasks such as multisensory decision-making or visual-spatial detection, where the stimulus is directly linked to behavior. By contrast, Dubreuil and Valente et al. reported that networks performing tasks that require flexible input-output mapping exhibit subcluster organization^40^. In line with this idea, the DMS-dr task in our study demands flexible output mapping based on combination of sensory inputs (e.g., the same first stimulus leads to different responses depending on the identity of the second stimulus). This requirement for dynamically integrating multiple inputs might promote the emergence of subcluster organization within the neural network.

Within the memory maintenance subspace, we observed that low and high tones were generalized to correspond with lick and non-lick responses, respectively (Fig.2j). This direction of generalization was consistent across animals and might reflect the sequence of task training. During the early stage of training, the mice were first exposed to low-low and low-high pairings, where the low tone during stimulus 2 was directly associated with the lick response. Although high-high and high-low pairings were subsequently introduced later during training, we speculate that the earlier training experience promoted an initial association between low tones and reward, causing low tones and lick behavior to become aligned along the same direction in the subspace. These findings suggest that the specific geometric features of memory maintenance representation are sculpted and may be associated with behavioral biases. This highlights a critical insight: rather than simply organizing information in a generalized geometric manner, the brain also encodes subtle, behaviorally relevant details within this generalized framework such as bias.

Our RNN simulations, constrained by empirical data, demonstrated the effect of lesioning specific functional modules in the mPFC (Fig.5). These findings align with previous theoretical studies^4,5^, reinforcing the concept of modular architecture and its reusability within the mPFC. Interestingly, silencing synaptic connections from the stimulus processing to the memory maintenance cluster during stimulus periods disrupted network behavior (Fig.5f to G). This suggests that in spite of the modular organization, the recurrent circuitry connecting these modules is necessary and sufficient for driving network dynamics. If the modular organization of these clusters in animal brains mirrors the architecture observed in our simulations, time-specific neural manipulations such as holographic perturbation^41–45^ could further uncover the underlying neural mechanisms of such architecture. Future work will be required to test this prediction and further elucidate the modular architecture and its reusability underlying flexible cognitive behavior.

In conclusion, our study provides evidence for clusters of neurons that are dedicated to specific cognitive computations. These computations can be reused across time and tasks. These findings suggest that reusable modular architecture underpins the complexity and flexibility of animal behavior. Furthermore, our results provide important insights into the significance of generalization in information representation, extending our understanding of how the brain supports this crucial cognitive function.

## Methods

### Mice

All procedures conducted in this study were approved by the Massachusetts Institute of Technology’s Animal Care and Use Committee and conformed to the Guide for the Care and Use of Laboratory Animals published by the National Institutes of Health. Male and female mice more than 8 weeks old were used in this study. Mice were housed in a room with reversed light/dark cycle (light off from 09:00 to 21:00) with controlled temperature and ventilation (20–22 °C; 40–60% humidity). All experiments were performed during the dark period of the cycle.

### Virus

For inactivation experiments, CaMKIIa-driven soma-targeted anion-conducting channelrhodopsin fused to FusionRed (pAAV-CKIIastGtACR2-FusionRed, Addgene, 105669; titre, 1 × 10^13^ viral genomes per ml) was used to express GtACR2 in the soma of excitatory neurons.

### Stereotactic surgeries

Animals were prepared similarly for all surgical procedures. Mice were anaesthetized using isoflurane anaesthesia (3% for induction, 1–1.5% for maintenance) while maintaining a body temperature of 37.5 °C using a heating pad (ATC2000, World Precision Instruments). Mice were given pre-operative slow-release buprenorphine (1 mg kg^−1^, subcutaneous injection) and post-operative meloxicam (1 mg/kg, subcutaneous injection). Mice were placed in a stereotaxic frame, their scalp hair removed, and the incision site sterilized using betadine and 70% ethanol. The skull was exposed and the conjunctive tissue removed using hydrogen peroxide. The skull was positioned such that the lambda and bregma marks were aligned on the anteroposterior and dorsoventral axes. For all surgeries, anti-inflammatory (Meloxicam) injections were pursued for 3 days following surgery.

For virus delivery, we first drilled a small craniotomy (0.5 mm) above the region of interest. For delivering virus in the mPFC or PPC, we injected a volume of 300–400 nl of virus (rate: 200 nl/min), using a glass pipette with a 50 μm diameter tip. Coordinates for targeting the mPFC/PPC virally were (in mm): anterior-posterior (AP) +1.5 to +2.0/−1.0, medial-lateral (ML): ±0.4/±1.5, dorsal-ventral (DV) – −1.75 and −2.25/ −0.5. We defined mPFC based on previous literature that included anterior cingulate cortex, prelimbic, and infralimbic cortex as part of PFC in rodents^46^. All injections were performed using an infuser system (QSI 53311, Stoelting) attached to the stereotaxic frame.

To deliver light into the mPFC or PPC, 200-μm two-ferrule cannulas (1.25mm ceramic ferrule 200 μm, Thorlabs, CFMLC12U-20) were implanted bilaterally above the mPFC or PPC using the following coordinates (in mm): mPFC: AP +1.5; ML: ±0.4; DV 1.85 at 10° in the ML axis; or PPC: AP: −2.0; ML: ±1.5; DV 0.4 at a 0° in the AP/ML axis. After implantation, dental cement (Teets Denture Material) and Metabond (C&B Metabond, Parkell) was applied to affix the implant to the skull. To avoid light reflection and absorption, the transparent Metabond was mixed with black ink pigment (Black Iron Oxide 18727, Schmincke). A custom designed head-plate was then positioned over the implant and affixed to the skull using Metabond. We used single ferrule cannulas with large (200 μm) diameter and high numerical aperture (0.39 NA) (Thorlabs, M89L01).

To perform mPFC and PPC single unit recording or optogenetic inhibition in awake head-fixed mice, we implanted a head plate parallel to the bregma–lambda axis of the skull. We used a custom design stereotactic arm to align the head plate parallel to the median and dorsal line of the skull during implantation. The head plate was attached to the skull using dental cement. The exposed skull was protected using rapid curing silicone elastomer (Kwik-Cast, WPI) topped with a fine layer of dental cement.

### Behavioral setup

Mice were head-fixed on a behavior rig and confined in a 3d-printed tube to limit body movements. A metallic lick spout position was controlled by mounting the spout apparatus on the 3d-printed stage controlled by miniature servo motor (Miuzei Mini Servo, MS18, China). The lick spout connected to a custom-made lick detector and was used to deliver water rewards (~10 μl drop of water). A small tube, pointing toward the mouse facial area and at a distance of 3 cm, was used to deliver air-puff punishment (compressed air at 40 psi for 0.3 s). Voltage signals from the transducer and lick detector were recorded through a microcontroller board (Arduino UNO Rev3). A second microcontroller board was used to control a 5 mm white LED light placed 8 cm in front of the mouse right eye, and two solenoid valves (Parker 003–0141-900) for water and air-puff delivery. Three or fourteen kilohertz sound stimuli of 0.3 s duration were delivered using a single speaker located at a distance of 30 cm from the mouse. The speaker frequency range was calibrated using a USB calibrated measurement microphone (UMIK-1, Mini DSP) and the Room EQ Wizard software. We used five behavior rigs (four for general behavior, optogenetics, and training, one for electrophysiological recording). The behavioral setup was connected to a computer running a custom-written MATLAB (Mathworks) script that was able to detect licks while controlling the timing of the light cue, sound (using custom-written MATLAB code), lick port position, water, and reward. Behavior rigs were assembled primarily with optomechanical components (Thorlabs).

### Behavioral task and training

Upon recovery from the surgical procedure, mice were gradually put on a water restriction schedule, receiving eventually 1–1.6 ml of water in total per day. Body weight was maintained above 90% of the pre-restriction weight.

A white LED indicated the beginning of each trial. After a 1–1.5s delay, two auditory stimuli (0.3s duration) separated by a first-delay epoch (1.0 s) were presented and followed by another second-delay epoch (1.0 s). After waiting until the delay 2 epoch, the lick spout was rapidly moved within reach of the tongue, and remained within reach for 1.0 s if licks were not detected. Mice learned to lick the spout when they heard two identical tones (matched trial, 3–3/14–14 kHz frequencies in first- and second-stimulus) and to hold still when they heard two different tones (non-matched trial, 3–14/14–3 kHz). Correct licks during this period were rewarded with ~10 μl drop of water and the spout remained within reach for an additional 2.0 s for licking water. Licks to the non-matched trial were punished with a siren-like auditory stimulus alone (early training) or siren plus air-puff (late training) and an extra 7.0 s inter-trial interval. At the end of the response epoch, the spout was then rapidly retracted and remained out of reach until the next trial (3–4s inter-trial interval).

Mice were taken through the following two stages of training until they became proficient at the task.

1. During the first phase of training (1st stage), mice learned to associate a lick with reward and to detect half category of matched/non-matched trials. In this phase, only 3–3 and 3–14 kHz pairs of tones were used. The delay 2 was set to 0.2 s and air-puff was not presented in this stage. Once mice performed successfully over 70% of trials in consecutive two days, they proceeded next stage.
2. During the second phase of training (2^nd^ stage), all pairs of tones were applied pseudo-randomly. The delay 2 was initially set to 0.2 s and air-puff was delivered only in false alarm trials. To prevent behavioral preference, an incorrect response resulted in the repetition of the same trial, thereby specifically increasing the trial length of the trial types with weak performance. Once they performed over about 100 trials, an incorrect trial was not repeated (test session). During the session, the performance correct rate (referred to as “correct rate” or “behavioral performance” in labels of figures) of each session was defined by: $Correct\;rate\;=\frac{Number\;of\;hit\;and\;correct\;rejection\;trials}{Total\;number\;of\;trials}$. Once mice performed successfully over 70% (65% for optogenetic inactivation batches) of trials in two consecutive days, the second-delay was prolonged by 0.1 s additionally in the next session.

### Optogenetic inhibition of mPFC and PPC activity

Optical stimulation was applied through a ferrule-terminated 200 μm core and 0.39 NA optic fibre attached to the 200 μm core and 0.39NA patch cable (Thorlabs, M89L01) using a 1.25mm ceramic mating sleeve (Thorlabs, ADAL1–5). We used a blue-fibre-coupled light emission diode (λ = 470 nm, Thorlabs, M470F3). The light was delivered at 20Hz with a 0.4 duty cycle at an irradiance of 10mW mm^−2^ at the output tip of the fibre.

We performed optical stimulation at one of six timings (0–0.3s, 0.4–0.7s, 0.8–1.1s, 1.3–1.6s, 1.7–2.0s, or 2.1–2.4s relative to first-stimulus onset) pseudo-randomly. Optical stimulation trials were randomly interleaved two times out of every ten trials (20% of trials), and were not applied in two consecutive trials. The optical stimulation was delivered to both hemisphere/cannulas simultaneously.

### Electrophysiology

For in-vivo electrophysiology recordings, expert mice were anesthetized with isoflurane (3% induction, 1–1.5% for maintenance). They underwent a 1mm craniotomy (centered at 1.5/−2.0 mm anterior to bregma and ±0.4/1.5 mm lateral to the midline for mPFC/PPC). The dura was punctured and the craniotomy was protected with saline and a piece of gel form (Pfizer). The skull was covered again with silicone (Kwik-Cast, WPI) and the mouse was allowed to recover for at least 24h for the anesthesia effect to wash out completely.

The awake animal was then head-fixed and the silicone and gel form removed gently. 0.9% NaCl solution was used to keep the surface of the brain wet for the duration of the recordings. After placing the animal in the recording setup, we submerged a reference silver wire in the saline solution on the skull surface. The position of the NeuroPixels 1.0 probe^29^ (IMEC, Belgium) was referenced on the surface of the brain. The probe was then lowered slowly (about 1 min per mm), by manually insertion using a micromanipulator (MP-285, Sutter Instrument Company), until a depth of 3.0 mm was reached. Electrophysiological data and time stamps of each trial start were collected at a rate of 30kHz with a PXI based system (National Instruments), and saved using OpenEphys software (Neuropixels PXI plugin, Open ephys, https://open-ephys.github.io/gui-docs/User-Manual/Plugins/Neuropixels-PXI.html). We recorded up to 6 sessions per mouse. Probes were dipped in CM-DiI, DiD, or DiO (Invitrogen, ThermoFisher, #V22889) prior to insertion. In each session, we inserted up to 2 probes at a time. The probes were always inserted at the same angle within the coronal plane (−10° and 10° relative to the vertical axis for mPFC and PPC) to aid subsequent histological probe tract tracing.

Spike sorting was done using Kilosirt2.5^47^, and spikes were manually curated using Phy GUI (https://github.com/kwikteam/phy) to remove artifacts. Units with an inter-spike interval (ISI) violation ≤ 0.5, firing rate ≤ 0.1 Hz, or ill-shaped waveform were filtered out. Spike times were verified with cross correlograms to combine units or eliminate duplicates. For each unit, part of the recordings with obvious drift (unit spikes abruptly disappearing) were excluded.

### Histology

All mice were deeply anesthetized and transcardially perfused with 0.1 M phosphate-buffered saline (PBS) followed by 4% paraformaldehyde (PFA). Brains were dissected and post-fixed in 4% PFA overnight at 4°C. Brains were then sectioned into 100 μm coronal sections using a vibratome (Leica VT 1200S) and mounted on SuperFrost Plus slides (VWR) with Vectashield mounting media (Vector Laboratories). Brain sections were imaged with a confocal microscope (Leica SP8) using a 10x objective to confirm probe and implant positions in target regions.

### Analysis of behavior

To quantify behavior, we calculated hit and false alarm rates defined as follows:

$$\mathrm{Hit}\;\mathrm{rate}=\frac{\mathrm{Number}\;\mathrm{of}\;\mathrm{hit}\;\mathrm{trials}\;}{\;\mathrm{Total}\;\mathrm{number}\;\mathrm{of}\;\mathrm{hit}\;\mathrm{and}\;\mathrm{miss}\;\mathrm{trials}\;}$$

$$\mathrm{False}\;\mathrm{alarm}\;\mathrm{rate}=\frac{\;\mathrm{Number}\;\mathrm{of}\;\mathrm{false}\;\mathrm{alarm}\;\mathrm{trials}\;}{\;\mathrm{Total}\;\mathrm{number}\;\mathrm{of}\;\mathrm{false}\;\mathrm{alarm}\;\mathrm{and}\;\mathrm{correct}\;\mathrm{rejection}\;\mathrm{trials}\;}$$

Discriminability (d’) was also used to quantify mice performance, as defined by:

$$d^{\prime }=\;\mathrm{norminv}(\mathrm{Hit}\;\mathrm{rate})\;-\;\mathrm{norminv}(\mathrm{False}\;\mathrm{alarm}\;\mathrm{rate})$$

where, norminv was the inverse of the cumulative normal function.

For behavioral data obtained from optogenetic inactivation experiments (Fig.1g), all behavioral metrics were calculated by pooling trials separately for mice injected with virus in the mPFC (n=3) and PPC (n=3), respectively. Statistical significance was assessed using a bootstrap test, performed by randomly sampling 50% of trials 1,000 times. Distributions were considered significantly dissociated if they showed no overlap within a 2SD range (containing 95.5% of data).

To quantify the impact of task and behavioral variables on behavior, we carried out regression analysis to weigh the contributions of stimulus and history of correctness, stimulus, false alarm, hit, and choice on the animal’s choice on the current trial^48^. To do so, we concatenated data from multiple sessions for each mouse and fit the animal’s choice with a logistic regression model as follows:

$$P(\mathrm{lick})=\mathrm{lapse}+\frac{1\;+\;2\mathrm{lapse}\;}{1\;+\;e^{-A}}$$

where

$$A=\beta_{0}+\sum_{t=1}^{T}\beta_{\mathrm{corr}}^{-t}C_{\mathrm{corr}}^{-t}+\sum_{t=1}^{T}\beta_{\mathrm{pro}-\mathrm{stim}}^{-t}P^{-t}+\sum_{t=1}^{T}\beta_{\mathrm{choice}\;}^{-t}C_{\mathrm{choice}}^{-t}+\sum_{t=1}^{T}\beta_{FA}^{-t}F_{FA}^{-t}+\sum_{t=1}^{T}\beta_{\mathrm{Hit}}^{-t}H_{\mathrm{Hit}}^{-t}+\beta_{\mathrm{stim}\;}S$$

where $\beta_{\mathrm{corr}}^{-t}$, $\beta_{\mathrm{pro}-\mathrm{stim}}^{-t}$, $\beta_{\mathrm{choice}\;}^{-t}$, $\beta_{FA}^{-t}$, $\beta_{\mathrm{Hit}}^{-t}$ and $\beta_{\mathrm{stim}}$ are coefficients of the $C_{\mathrm{carr}}^{-t}$, $P^{-t}$, $C_{\mathrm{choice}}^{-t}$, $C_{FA}^{-t}$, $C_{\mathrm{Hit}}^{-t}$, S regressors, respectively. T is the number of trials in the past. For this model, we took into account for up to 3-trial history. In this equation (5), $C_{\mathrm{corr}}^{-t}$, $F_{FA}^{-t}$, $H_{\mathrm{Hit}}^{-t}\in [+1,\;0]$ if a trial is correct or incorrect, false alarm or not, and hit or not at t-back trial, $P^{-t}\in [+1,-1]$ if a trial is a matched or non-matched at t-back trial, $C_{\mathrm{choice}}^{-t}\in [+1,-1]$ if the mice licked or non-licked a port at t-back trial, S∈[+1,-1] if the current trial is a matched or non-matched, respectively. $\beta_{0}$ is the intercept of the model that captures the overall bias of mice. We used the negative log-likelihood as the cost function J:

$$J=-(y\;log(p)+(1-y)\;log(1-p))=-[\sum_{i}^{m}y_{i}log\left(p_{i}\right)+\left(1-y_{i}\right)\;log\left(1-p_{i}\right)]$$

where m is the total number of trials. The model was fit using a gradient-descent algorithm to minimize the negative log-likelihood cost function with elastic net regularization with parameters α=0.2 (L1) and λ=0.8 (L2). We used the sqp algorithm in the *fmincon* function from MATLAB. To compare the impact of parameters of current stimulus and previous trial information, we also fit the model without current stimulus (S) or previous trial parameters ($C_{\mathrm{corr}}^{-t},\;P^{-t}$, $C_{\mathrm{choice}}^{-t}$, $F_{FA}^{-t}$, and $H_{\mathrm{Hit}}^{-t}$ ). All models were fit using 200 runs of fivefold cross-validation. For each run, we computed the log-likelihood for the test dataset given the best-fit parameters on the training set (logl). We also calculated the log-likelihood of the test dataset given the only best-fit parameters of $\beta_{0}$. This gives us a null log-likelihood reference value ($logl_{0}$). In order to quantify the efficiency of each model we defined the cross-validated bit/trial (CV-bit/trial) as the trial-averaged excess likelihood of the model compared to the null model^48^:

$$\frac{\left(log\;l\;-\;log\;l_{0}\right)/n_{\mathrm{trials}}}{log\;2}$$

To compare different models, we calculated the mean value of CV-bit/trial across 200 runs for each mouse.

### Analysis of neural activity

For all mPFC and PPC neurons, changes in firing rate associated with behavior were assessed using peri-stimulus time histogram (PSTHs). Unless otherwise stated, PSTHs were computed using 10 ms bins for individual neurons in each recording session and smoothed with a Gaussian filter with a standard deviation of 20 ms to obtain the temporal profile. For visualization and analysis, firing rates were z-scored across sessions.

#### Selectivity

All comparative indices (stimulus 1, 2, and choice selectivity index) were computed using a receiver operating characteristic (ROC) analysis (Fig.1h and Supplementary Fig.2), which calculates the ability of an ideal observer to classify whether a given spike density was recorded under one of two trial types. We indexed the difference between two firing rate distributions by scaling the ROC area between −1 and 1, where 0 reflects no difference between the distributions and the sign denotes whether a neuron fires more under one of the trial types than the other. Statistical significance (p<0.05) was determined with a permutation test of 500 repetitions. In this analysis, we used firing rates in only correct trials during the task.

We assessed stimulus 1 and 2 selectivity by comparing high and low tone trials at the stimulus (0–0.3s and 1.3–1.6s relative to stimulus 1 onset) or delay period (0.8–1.3s and 2.1–2.6s relative to stimulus 1 onset). Choice selectivity was computed by comparing lick and non-lick trials at the delay period.

#### State-space analysis

For state-space analysis (Supplementary Fig.3), we used neurons that were recorded in the sessions with >65% correct rate. To characterize the population structure and temporal pattern among all neurons during the analysis window (stimulus subspace: 0–0.5s and 1.3–1.8s relative to stimulus 1 onset, memory maintenance subspace: 0.8–1.3s and 2.1–2.6s), z-scored firing rates were formatted as $X\in R^{N\times CT}$, where N is the number of neurons, C is the total number of conditions (e.g., high/low tones in stimulus 1 and 2 period), and T is the number of analyzed time points. Principal component analysis (PCA) was used to reduce the dimensionality of the population from the number of neurons to ten principal components (PCs). Each PC represents a weighted combination of individual neuronal activity, which summarizes population activity.

To statistically estimate the difference between each neuronal trajectory at each time point across trial types in the PC space, we computed each PC using a subset of trials (50%) and projected the data from the remaining subset of trials (50%) onto each axis. The projection of each axis was computed by the dot product as $v_{pc}x$, where $v_{pc}$ is a weight vector for each PC, and x is an N×(4×time) matrix of smoothed, trial-averaged firing rates across trial types (low-low, low-high, high-low, and high-high). The procedure was repeated 100 times with shuffling trials within each trial type. We then computed the Euclidean distance between low and high tone trials (stimulus) or lick and non-lick trials (choice) using the first 3 or 10 dimensions. Statistical significance was tested by comparing the resampling distributions of distance between empirical and shuffled-label data. If the distributions were not overlapped in a 2SD range (95.5% data in this range), they were defined as significantly dissociated.

#### Generalized linear model (GLM)

We used a GLM to determine how various task variables affect the responses of each neuron (Supplementary Fig.6). We first z-scored the responses of a given neuron by subtracting the mean response from the firing rate at each time and in each trial and by dividing the result by the standard deviation of the responses. Both the mean and the standard deviation were computed by combining the neurons responses across all trials and times. We then described the z-scored responses of neuron i at time t as a linear combination of several task variables (Supplementary Fig.6a):

$$fr_{i}(t)=\beta_{\mathrm{stim}1}(t)Stim1+\beta_{\mathrm{stim}2}(t)Stim2+\beta_{\mathrm{choice}}(t)\;\mathrm{Choice}\;+\beta_{0}(t)+\varepsilon (t)$$

where $fr_{i}(t)$ is the z-scored response of neuron i at time t,Stim1 indicates the category of stimulus 1 (+1: low tone; −1: high tone), Stim2 indicates the category of stimulus 2 (+1: low tone; −1: high tone), and Choice represents the animal’s choice (+1: licked; 0: non-licked). $\beta_{0}(t)$ and ε(t) denote the intercept and residual of the z-scored response, respectively.

To estimate the optimal weights for each variable ($\beta_{\mathrm{stim}1}(t)$, $\beta_{\mathrm{stim}2}(t)$, $\beta_{\mathrm{choice}}(t)$, and $\beta_{0}(t)$) while preventing overfitting, the GLMs were fitted to each neuron’s response using the *lassoglm* function in MATLAB with five-fold cross validation of the training data. We specified a normal distribution with a linear (identity) link function and set the hyperparameter λ to 0.01. Only neurons recorded in the sessions with >70% correct rate were included in the GLM analysis.

For assessing the significance of pairwise correlations, we computed Pearson’s correlation coefficients (r) and their associated *p*-values using the *corr* function in MATLAB. The function calculates two-tailed p-values under the null hypothesis of no correlation (r=0) by transforming the correlation coefficient into a t-statistic.

#### Classification analysis

For classification analysis (Fig.2c, f, Supplementary Fig.4a–c, Supplementary Fig.5a–c, and Supplementary Fig.5e–g), we used neurons that were recorded in the sessions with >65% correct rate. For classifiers, we used a logistic regression classifier as implemented by the MATLAB *fitclinear* function to quantify the amount of information about the stimulus (low or high tone) and choice (lick or non-lick trial) in the population of neurons recorded from each brain region. This analysis included all neurons that were recorded during at least 40 trials for each stimulus and choice trial types, and we constructed “pseudo-trials” by randomly extracting trials from desired conditions for each neuron^49^. For the training and testing dataset, the number of trials in each condition was matched to prevent bias in training classifiers. We also randomly chose the trials of lick trials from low-low and high-high trials and non-lick trials from low-high and high-low trials with equal probability (20 trials for each combination). Classifiers with L2 regularization (λ = 1/20) was trained to classify the stimulus or choice using pseudo-population data and tested on held-out data. The choice of the λ value did not affect our conclusion. We used tenfold cross-validation by leaving a 10% subset of trials for classification to avoid overfitting. This procedure was repeated 100 times. Classification results are reported either as a function of time or in a fixed time window. Time-resolved classification was done on z-scored firing rates measured in a 50 ms moving window. For fixed-window classification, we used z-scored firing rates in a 500ms for stimulus (0–0.5s or 1.3–1.8s from stimulus onset) and delay (0.8–1.3s or 2.1–2.6s from stimulus onset) periods. For cross-conditional classification approach, we trained a classifier to classify one condition (e.g., high/low tone trials) using a pseudo-population data from the epoch/time corresponding to the condition (e.g., delay 1 epoch), and tested on held-out data from another epoch/time corresponding to another condition (lick/non-lick in delay 2 epoch). Statistical significance was tested by comparing the resampling distribution of classification accuracy averaged within tenfold cross-validation against chance level (chance level = 0.5 for stimulus and choice classification). If the distribution and the chance level are not overlapped in a 2SD range (95.5% data in this range), they are defined as significantly dissociated.

#### Angle between subspaces

For calculation of angle between axes (Fig.2d, e, g, h, Supplementary Fig.4c, Supplementary Fig.5f), the decision boundary of a logistic regression classifier can be computed as

$$w^{T}z+b$$

where w is the weights, z is z-scored firing rates, and b is an intercept. We obtained $w^{\mathrm{cond}1}$ and $w^{\mathrm{cond}2}$ for each condition and computed angle θ between these as:

(1) $$\theta ={cos}^{-1}\frac{W^{\mathrm{cond}1}\;\cdot \;W^{\mathrm{cond}2}}{\left|W^{\mathrm{cond}1}\right|\left|W^{\mathrm{cond}2}\right|}$$

The angle θ was converted from radian to degree ranged 0 to 90 degrees. Statistical significance was tested by comparing the resampling distribution of empirical angles and angles computed based on the weights obtained from classifiers trained with shuffled-label data.

For calculation of angle between axes for each animal (Fig.2e, h), we first calculated the condition-averaged z-scored firing rate (high/low, or lick/non-lick) for each neuron for the relevant epoch. To obtain the “Coding Direction^50,51^”, we computed the difference of firing rates between conditions. Specifically, we defined the following two axes for this analysis. The “stimulus axis” was computed from the dissociation of neuronal activity between the low and high tone trials in the stimulus period (0–0.5s, or 1.3–1.8s from first-stimulus onset). The “memory maintenance axis” was computed from dissociation of neuronal activity between low and high tone trials or lick and non-lick trials in the latter-half of delay period (0.8–1.3s, or 2.1–2.6s from first-stimulus onset). We prepared the four vectors, which are average population activity v of length $N_{\mathrm{unit}}\times 1$. Each vector was normalized by their unit length as $v_{\mathrm{norm}}=v/\|v\|$. The angle between stimulus axes was calculated as equation (1) using $v_{\mathrm{norm}}$ calculated using stimulus periods. The angle between memory maintenance axes was calculated using $v_{\mathrm{norm}}$ from memory periods.

The significance of association between behavioral performance and the angle of subspaces was assessed by a correlation test (Fig.2e, h, and Supplementary Fig.5d, P<0.05, Spearman rank-correlation test).

#### Geometric analysis

For geometric analysis (Fig.2i–j), we used neurons that were recorded in the sessions with >65% correct rate. To visualize the geometry of trials in the population activity, z-scored firing rates were formatted as $X\in R^{N\times 8}$, where N is the number of neurons and four combinations of tones (low-low, low-high, high-low, and high-high tones) at two stimulus periods (0–0.5, and 1.3–1.8s from stimulus 1 onset) or the latter-half of delay periods (0.8–1.3, and 1.8–2.3s from stimulus 1 onset). PCA was used to reduce the dimensionality of the population from the number of neurons to a lower number of principal components (PCs).

To study how the geometry of the first-two PC space evolved throughout the trial, the projections of the first-two axes were computed as the same procedure described in state-space analysis at each time throughout the trial (Supplementary Fig.7).

#### Clustering analysis

To examine the dominance of specific clusters of neurons contributing to the stimulus and memory maintenance subspace, we conducted clustering analysis based on neurons’ response profile to the task variables (Fig.3c–g). First, we assume that we have recorded a response profile $R\in R^{N\times q}$, where N is the number of neurons and q is the number of features. The features were defined as the average firing rate during four different epochs (0–0.5s, 0.8–1.3s, 1.3–1.8s, and 2.1–2.6s from stimulus 1 onset) for each unique combination of tones, resulting in 16 features in total (Fig.3a). Second, we applied the GMM to the response profile matrix R. To do that, we used the *fitgmdist* function in MATLAB with a 0.35 regularization value, 100 replicates, and the covariance matrix constrained to diagonal^52^.

To determine the number of clusters, we computed the Bayesian information criteria (BIC) score (Fig.3e). It is a penalized likelihood term defined as $2\left(N\;\log\;L\right)+M\;log\;n$, where NlogL is the negative log-likelihood of the data, M is the number of parameters of the GMM and 𝑛 is the number of observations. The BIC score was computed by the *fitgmdist* function.

#### ePAIRS

To statistically test whether mPFC and PPC neurons exhibited clusters of prototypical response profiles or a uniform continuum of response profiles, we carried out the ePAIRS statistical test^24,40^ (Fig.3b, d), which is itself derived from the PAIRS test developed in ^22^. We used a response profile R described above as a representational space and it was z-scored for each neuron. For each neuron, we computed the median vector angle $\alpha_{i}$ with its k-nearest neighbors (k being a hyperparameter set to 3 in this study), defining an empirical distribution ${\overset{ˆ}{p}}_{\mathrm{data}}(\alpha )$. For comparison, we generated null distributions that exhibited no clustering. A multivariate Gaussian distribution N(0,Σ) is fit to the response profile R using *mvnrnd* function in MATLAB, with ∑ being the empirical covariance of R, computed as $\sum \;=\frac{1}{N}R^{T}R$. We then computed the median vector angle of nearest neighbors for this simulation dataset, defining a null distribution $p_{\mathrm{null}}(\alpha )$. The difference between the $p_{\mathrm{data}}$ and $p_{\mathrm{null}}$ is assessed using a two-sided Wilcoxon rank-sum test^40^.

### RNNs

We built a rate-based RNN where each unit’s activity matched the PSTH of an experimentally recorded neuron from the mPFC, pooled across sessions^53,54^ (Fig.5). All networks are a time-discretized RNN with positive activity. Before time discretization, the network state h evolves according to a dynamical equation:

(2) $$\tau \frac{dh}{dt}=-h+\varphi \left(W_{\mathrm{rec}}h\right)+W_{\mathrm{in}}u+\xi$$

Here, τ is the units’ time constant. φ(⋅) is the non-linear activation function, the softplus function was used in this study, $W_{\mathrm{rec}}$ and $W_{\mathrm{in}}$ are the recurrent and input weight matrices, u is the inputs to the network, and ξ represents Gaussian noise.

The network’s output units z was read out from the network according to:

$$z=W_{\mathrm{out}}h$$

where $W_{\mathrm{out}}$ is the output connection matrix of size $N_{\mathrm{out}}\times N_{\mathrm{rec}}$. All weight matrices ($W_{\mathrm{in}},W_{\mathrm{rec}},W_{\mathrm{out}}$) were learned over the course of training.

After using the first-order Euler approximation with a time-discretization step Δt, we have

$$h_{t}=(1-\alpha )h_{t-1}+\alpha \left(\varphi \left(W_{\mathrm{rec}}h_{t-1}\right)+W_{in}u_{t}\right)+\xi_{t}$$

Here, α≡Δt/τ, and we use a discretization step Δt=20ms. We imposed no constraint on the sign or the structure of the weight matrices $W_{\mathrm{in}}$, $W_{\mathrm{rec}}$, $W_{\mathrm{out}}$. The network and the training are implemented in PyTorch.

The network received three types of noisy input:

$$u=\left[u_{\mathrm{high}},u_{\mathrm{low}},u_{fix}\right]+u_{\mathrm{noise}}$$

$$u_{\mathrm{noise}}[j]\sim 0.03N(0,1)$$

The fixation input $u_{fix}$ was 1 when the network was required to fixate and 0 when the network was required to respond. The stimulus inputs ($u_{\mathrm{high}}$ and $u_{\mathrm{low}}$) represented high and low tones in the mouse’s experiment, respectively, and were set to 1 during the stimulus epoch and 0 otherwise.

#### Task and performance

We implemented an abstraction of the DMS-dr task that mice performed in this study. The task consisted of six periods; initial delay (100 ms), stimulus 1 (300 ms), delay 1 (1000 ms), stimulus 2 (300 ms), delay 2 (1000 ms), and response period (200 ms). Inputs and outputs for an example trial are shown in Fig.5a. Fixation input remained 1 throughout the trial until the response period, where it changed to 0. If the target fixation output prematurely fell below 0.5, the network was deemed to have erroneously broken fixation, resulting in an incorrect trial. Fixation input and output reflect the suppression of licking behavior in the mouse’s experiment. Stimulus inputs represented high and low tones in the experiment. The average of response output during the response period determined whether the trial was reported as a match (output>0.5) or non-match (output≤0.5), reflecting licking or non-licking behavior in the mouse’s experiment. The task performance was computed by comparing the response output with successfully reported with match and non-match trials.

#### Network training procedure

We used back-propagation through time^55^ to train networks to minimize loss functions L. Trials were generated stochastically, following a predefined temporal structure and mapping stimulus inputs to target outputs $\overset{ˆ}{z}$. The total loss function was:

(14) $$L=L_{\mathrm{out}}+L_{\mathrm{rate}}$$

where

(15) $$L_{\mathrm{out}}=\sum_{k,t}M_{k,t}{\left(z_{k,t}-{\overset{ˆ}{z}}_{k,t}\right)}^{2}$$

(16) $$L_{\mathrm{rate}}=\sum_{i,t}{\left(r_{i,t}-{\overset{ˆ}{r}}_{i,t}\right)}^{2}$$

Here, $z_{k,t}$ and ${\overset{ˆ}{z}}_{k,t}$ are, respectively, the actual and the target readout values and the indices k and t, respectively, are the index of the output and timesteps. $r_{i,t}$ and ${\overset{ˆ}{r}}_{i,t}$ are, respectively, the actual and the trial-averaged mPFC neuronal activity of the i-th neuron. We implemented a mask, $M_{k,t}$, for modulating the loss with respect to certain time intervals.

- For the response output unit (k=1), $M_{1,t}=0$ before stimulus 1 onset, $M_{1,t}=5$ during the response period, and $M_{1,t}=1$ for the rest of the trial.
- For the fixation output unit (k=2), $M_{2,t}=0$ before stimulus 1 onset and $M_{2,t}=2$ for all other timepoints.

We trained networks with N=312 and N=77 units to reproduce neural activity recorded from mPFC clusters 2 and 3 (Fig.4a), respectively. Additionally, we included other 61 neurons which were not trained to reproduce neuronal activity recorded from the mPFC. The total number of units in the network was N=450. The networks were trained using Adam optimizer^56^ in PyTorch with the decay rate for the first and second moment estimates of 0.9 and 0.999 and learning rates of 10^−2^, respectively. During training, we used mini-batches of 32 trials. In a given minibatch, all trial types were randomly generated. Training was terminated when $L_{\mathrm{out}}$ fell below 0.02, and the task performance exceeded over 0.99.

#### Unit inactivations

We silenced a network unit by setting its activity to zero for all specific cluster of neurons at given timepoints. Units were silenced at five epochs: initial delay (0–100ms from trial start), stimulus 1 (100–500ms), late-delay1 (900–1400ms), stimulus 2 (1400–1800ms), and late-delay 2 (2200–2700ms) (Fig.5c).

## Supplementary Material

Supplement 1

## Data Availability

All data and code supporting the findings from this study are available upon reasonable request from the corresponding author (M.S.).
