# Supplementary material for: Reusable modular architecture enables flexible cognitive operations
Source: Res Sq. 2025 Sep 19:rs.3.rs-7420993. Preprint. [Version 1] doi: 10.21203/rs.3.rs-7420993/v1 (PMC12458543; doi:10.21203/rs.3.rs-7420993/v1)
Supplement: Supplement 1 [file NIHPPRS7420993V1-supplement-1.pdf]

Supplemental Figures

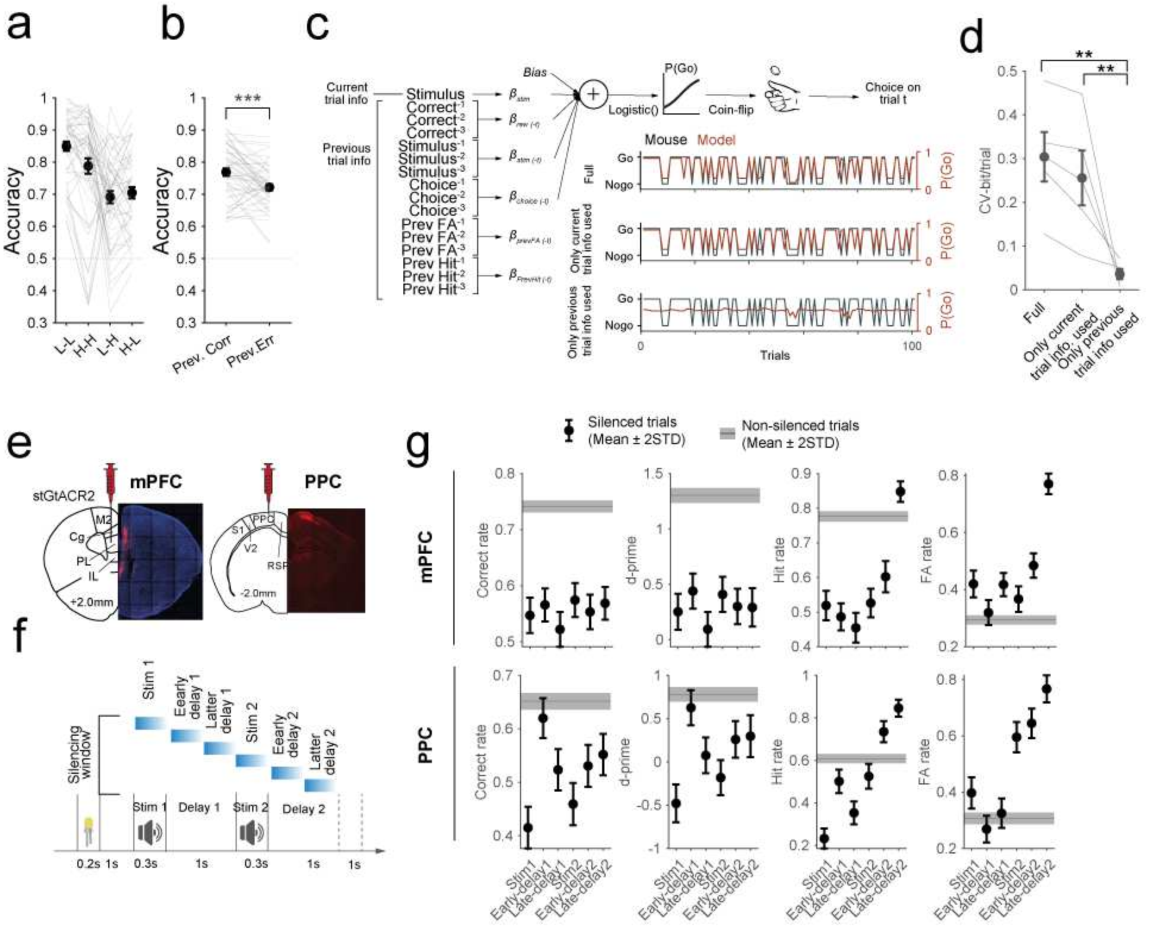

Extended Data Fig.1 | Behavioral metrics and probabilistic logistic regression of behavior

(a) Choice accuracy across all four tone-pairs trial types showing mice performed well on all trial-types (n = 53 sessions). Gray lines indicate individual mice, and the black line represents the group average.

(b) Comparison of choice accuracy following correct versus error trials showing significant behavioral performance improvement after correct trials (p<0.001, two-sided, paired Student's t-test). Gray lines represent individual mice, and the black line indicates the group average.

(c) A linear weighted sum of 16 task and behavioral variables was used to predict the log probability ratio $\log(P_{lick}/P_{non-lick})$. Beta weights were fit to the training data and evaluated on left-out cross-validation data (see Methods). Task and behavioral variables included: matched/non-matched stimuli, and correctness, stimulus, and choice on the last 1-3 trials. Bottom right: example mouse behavior and model performance based on different variables. Black and orange lines indicate mouse behavior and model prediction.

(d) Evaluation of model variants with different task and behavioral variables (Methods). Model performance using only the current stimulus was significantly better than when using only previous trial information.

(e) Schematic from Paxinos and Franklin illustrating the injection target and a histological section after the stGtACR2 showing injection localization.

(f) Schematic illustrating timing of photoinhibitions.

(g) Photoinhibition effect on the behavioral performance. Line and shaded area indicate the mean ± 2SD of non-silenced trials. All error bars indicate the mean ± 2SD.

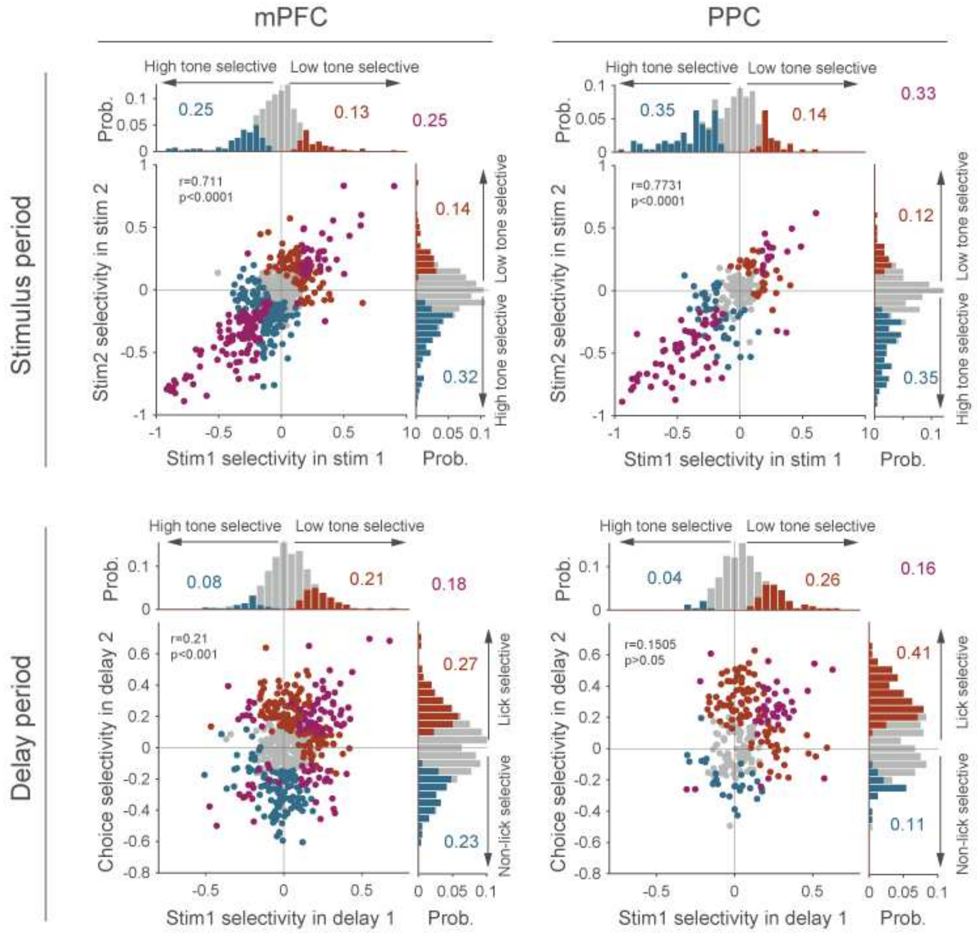

Extended Data Fig.2 |Neuronal selectivity in mice performing a DMS-dr task

Scatter plot and histograms showing stimulus and choice modulations for mPFC and PPC neurons. In both mPFC and PPC, neurons that exhibited selectivity for either the high tone or low tone during stimulus 1 maintained the same selectivity for the corresponding tone during stimulus 2 (top row, r=0.711 and 0.773, $p<0.001$ and $p<0.001$, for mPFC and PPC, respectively). However, in only the mPFC, neurons that exhibited selectivity for stimulus 1 during the delay 1 period also selectively responded to the planned motor response in the delay 2 period (bottom row, r=0.210 and 0.151, $p<0.001$ and $p>0.05$, for mPFC and PPC, respectively). These results suggest the reuse of single neurons for memory maintenance across task periods in mPFC. Colored bars in the histograms indicate neurons significantly modulated by stimulus or choice variables. Colored dots in the scatter plot demarcate neurons with significantly positive or negative modulation by stimulus, choice, or both (purple).

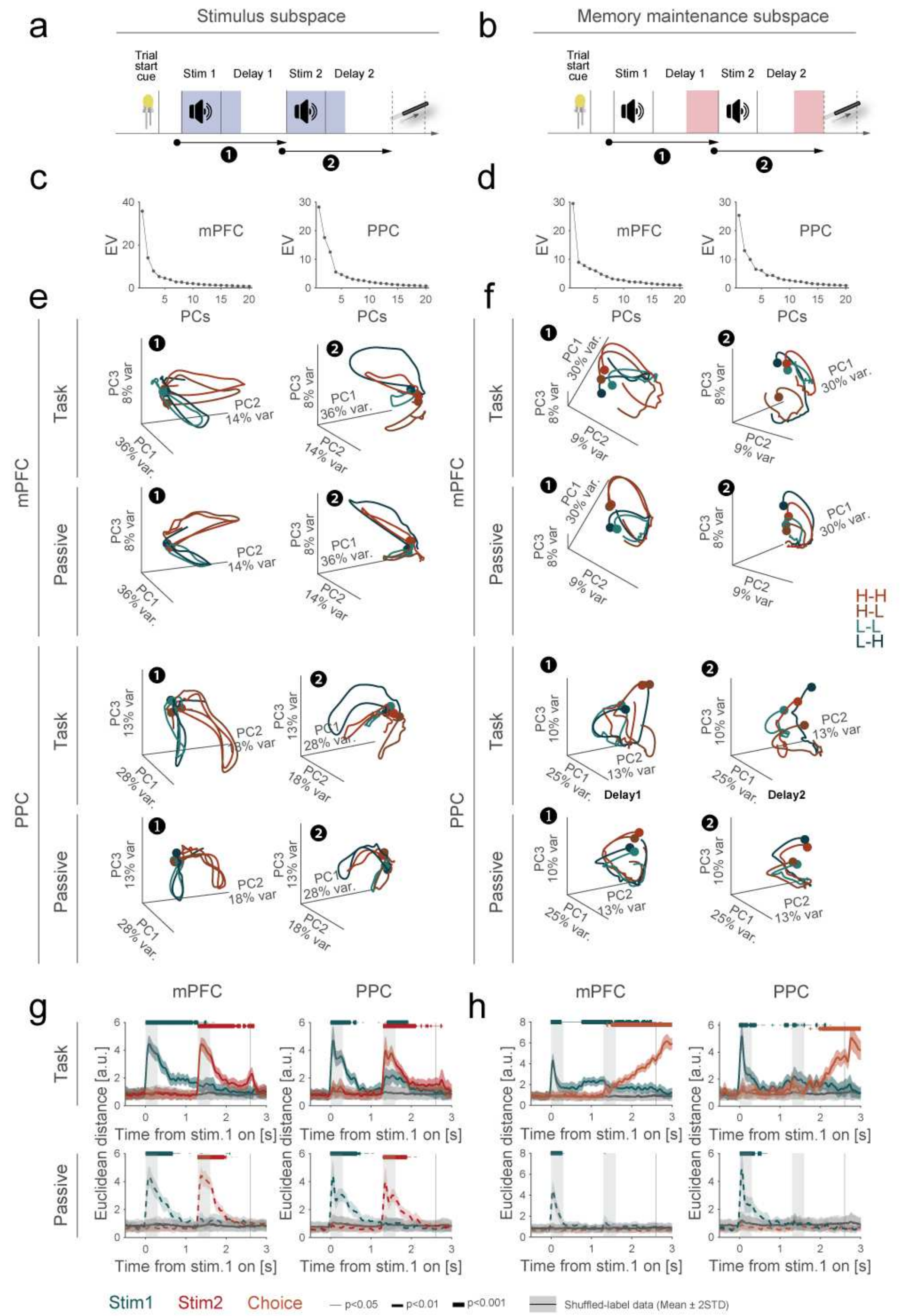

Extended Data Fig.3 | Population analysis for determining whether mPFC and PPC encode stimulus and choice information during active task and passive hearing sessions

(a-b) Schematics illustrating how the stimulus and memory maintenance subspaces are defined from population

activity in different task epochs. We applied a dimensionality reduction technique, PCA, to the trial-averaged responses of all neurons pooled across experiments ("pseudo-population") to define stimulus and memory maintenance subspaces. The stimulus subspace is defined from population activity during stimulus epochs (shaded area in **a**, [0 0.5] s and [1.6 2.1] s from stimulus onset), while the memory maintenance subspace is defined from late-delay epochs (shaded area in **b**. [0.8 1.3] s and [2.1 2.6] s from stimulus onset). The arrow at the bottom of the task timeline represents the projection window for all trials described in **e** and **f**.
(c-d). Explained variance of population activity during stimulus (**c**) and late-delay epochs (**d**) in the mPFC (left) and PPC (right).
(e-f) Neuronal population activity projected into the first three-dimensional space during task (top) and passive (bottom) blocks in the mPFC and PPC.
(g) Euclidean distance between low and high tones trials for stimulus 1 (blue) and stimulus 2 (red) during task (solid line) and passive (dashed line) blocks.
(h) Euclidean distance between low and high tones trials for stimulus 1 (blue), and between lick and non-lick trials (orange) during task (solid line) and passive (dashed line) blocks. These results suggest that population dynamics related to information maintenance during the late-delay epochs requires task engagement. Conversely, stimulus response dynamics may reflect bottom-up activity during the stimulus epochs. Together, both brain regions encode stimulus and choice information during the task at the population level. Shaded areas represent ± 2STD (95.5% of the data). Horizontal bars at the top of **g** and **h** indicate above-chance Euclidean distance (P < 0.05, 0.01, and 0.001 for thin, medium, and thick lines, respectively; bootstrap). All black lines and shaded area represent shuffled data.

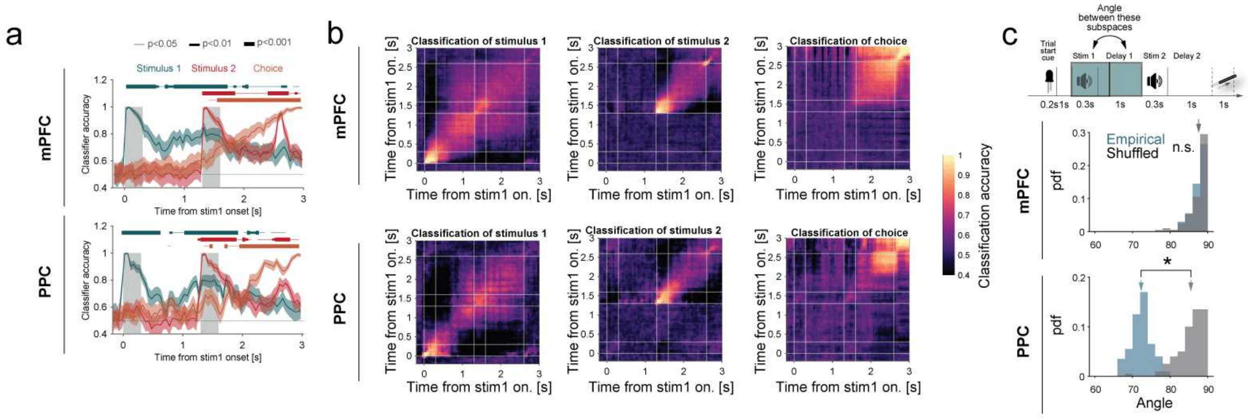

Extended Data Fig.4 | Neural subspaces of stimulus 1, stimulus 2, and choice during task and passive hearing sessions
(a) Time course of classifier accuracy for stimulus 1, stimulus 2, and choice information in the mPFC and PPC. Horizontal bars (top of each plot) indicate above-chance classification (P < 0.05, 0.01, and 0.001 for thin, medium, and thick lines, respectively; bootstrap test)
(b) Cross-temporal classifier accuracy for stimulus 1, stimulus 2, and choice information in the mPFC (N=12 sessions) and PPC (N=7 sessions).
(c) The angle between subspaces for stimulus 1 trained during stimulus and late-delay 1 periods. No significance between empirical and shuffled data in the mPFC indicates that these subspaces are orthogonal to each other.

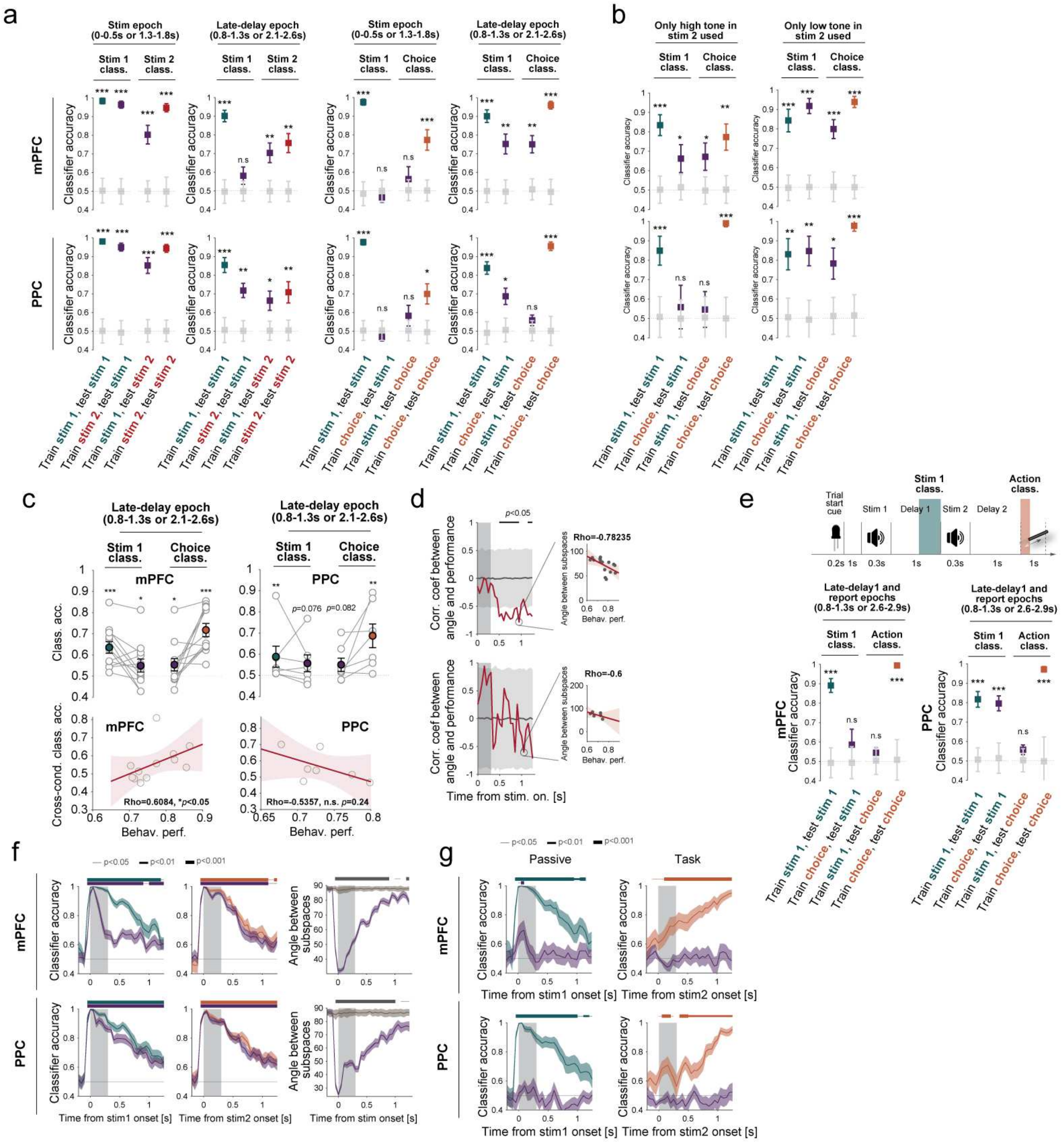

Extended Data Fig.5 | Shared subspaces of stimulus 1, stimulus 2, and choice during task and passive hearing tasks

(a) Classifier accuracy for stimulus 1, stimulus 2 and choice information during stimulus and late-delay epochs. Blue, red, and orange bars indicate classification accuracies for stimulus 1, stimulus 2 and choice, respectively. Purple bars indicate cross-conditional classifier accuracy, and light gray bars indicate classifier accuracy on shuffled-label data. All classifier accuracies are shown as mean ± 2STD.

(b) Same as e of late-delay epoch but only low tone (left, L-L and L-H) or high tone (right, H-L and H-H) in stimulus 1 was used.

(c) (top) Classification accuracy for stimulus 1 and choice during late-delay epoch for each session for mPFC and PPC. (bottom) Cross-conditional classification accuracy as a function of behavioral performance. Each point indicates each session.

(d) Correlation coefficients between behavioral performance and stimulus-choice subspaces angle during late-delay

epochs over time.
(e). Classifier accuracy of stimulus 1 and action (lick or non-lick trials) during late-delay1 and action period, respectively.
(f). (Left) Time course of classifier accuracy for stimulus 1 and stimulus 2 information during the passive block for each region. (Right) Time course of the angle between stimulus1 and stimulus 2 subspaces for each region during passive block.
(g) Time course of classifier accuracy for stimulus 1 information during the passive block and choice information during the task block. Horizontal bars at top of each plot of the time course of classification accuracy indicate above-chance classification (P < 0.05, 0.01, and 0.001 for thin, medium, and thick lines, respectively; bootstrap).

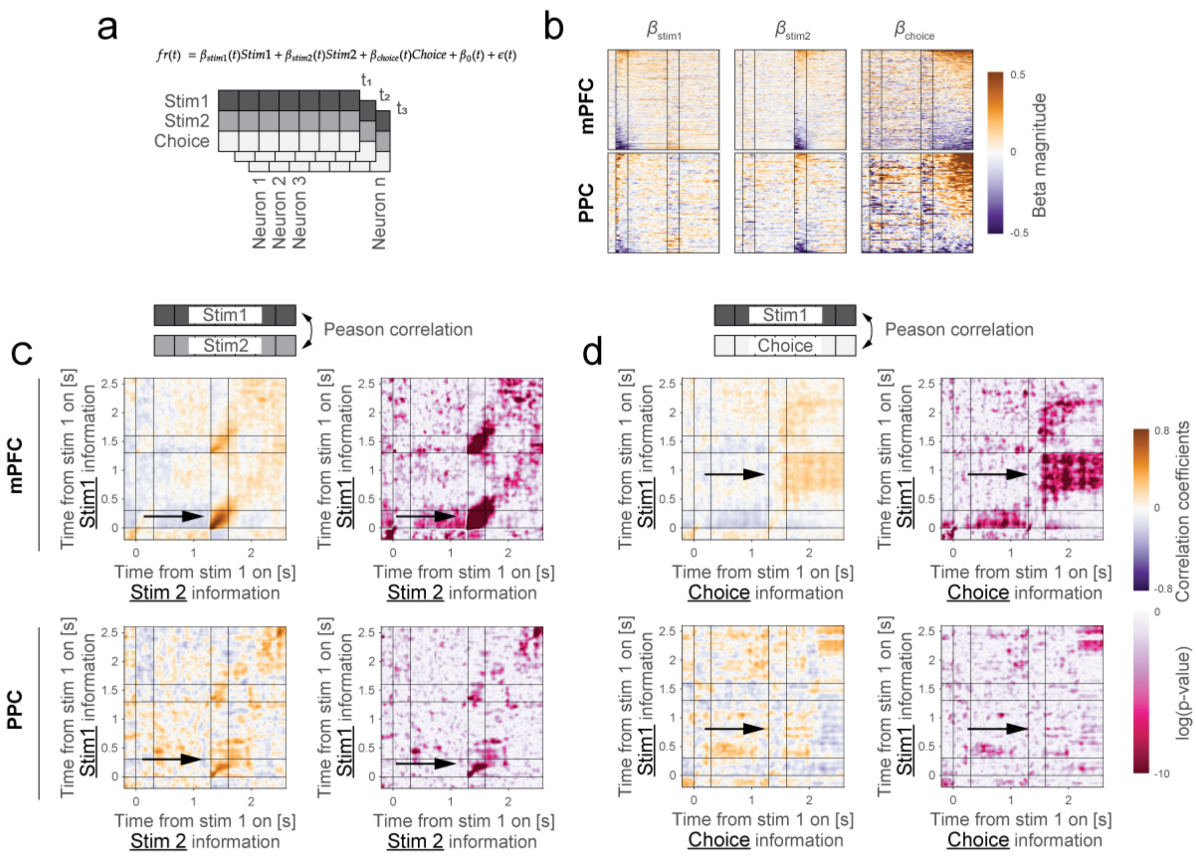

Extended Data Fig.6 | GLM analysis for determining whether the mPFC and PPC share the subspaces for stimulus and choice during task
(a) Schematic of the beta coefficients used in the GLM analysis.
(b) Temporal dynamics of GLM weights for stimulus 1, stimulus 2 and choice in the mPFC and PPC. Neurons are sorted by their beta magnitude during stimulus 1, stimulus 2, and delay 2 periods for stimulus 1, stimulus 2, and choice betas.
(c) Cross-temporal correlation coefficients for stimulus 1 and stimulus 2 in the mPFC and PPC (right), along with corresponding p-value (left) (Methods). We found significant correlations between stimulus 1 and stimulus 2 during stimulus presentation in both the mPFC and PPC (arrow). This indicates that there is a shared subspace between them.
(d) Same as **c**, but for stimulus 1 and choice. Only the mPFC shows significant correlations between stimulus 1 and choice during the delay (arrow). This indicates that a shared subspace for memory maintenance only exist in the mPFC.

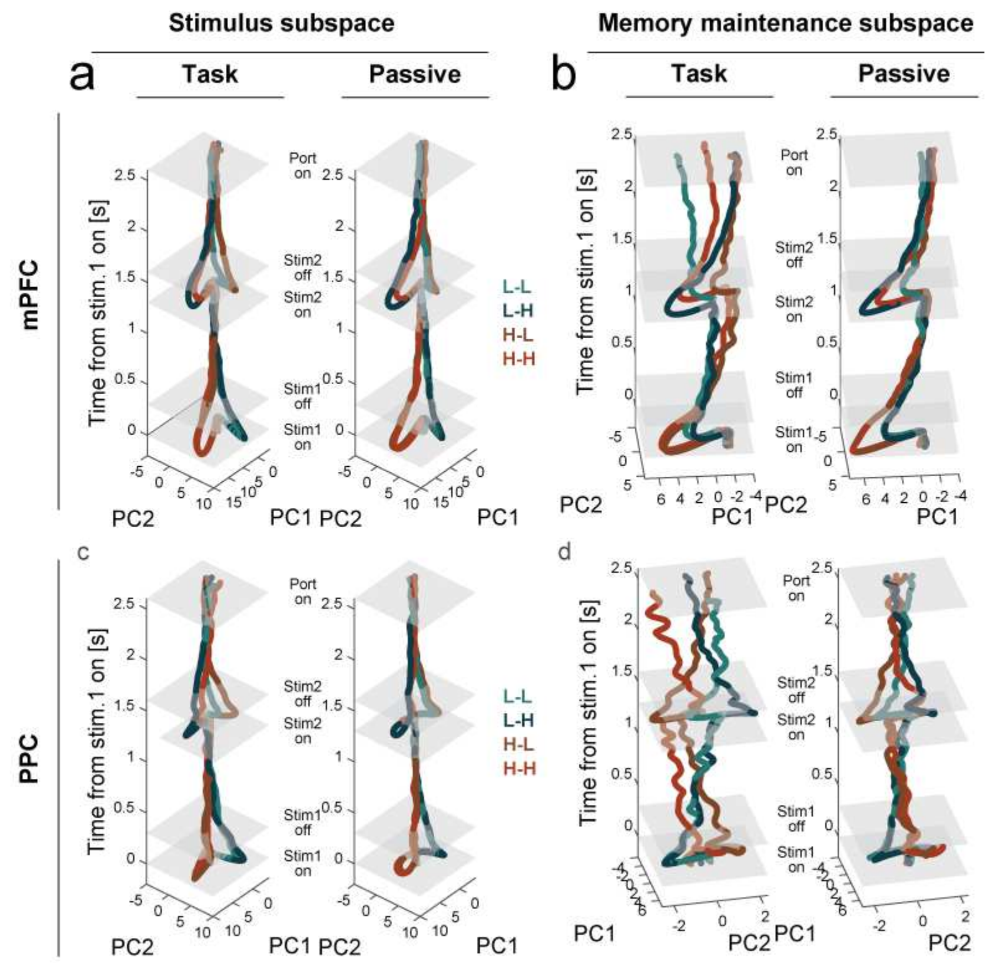

Extended Data Fig.7 |Evolution of neural geometry of stimulus and memory maintenance subspaces
(a-b) Trial representations in the mPFC and PPC projected onto the stimulus subspace over time during the task (left) and passive (right) blocks.
(c-d). Same as **a** and **b**, but for memory maintenance subspace.

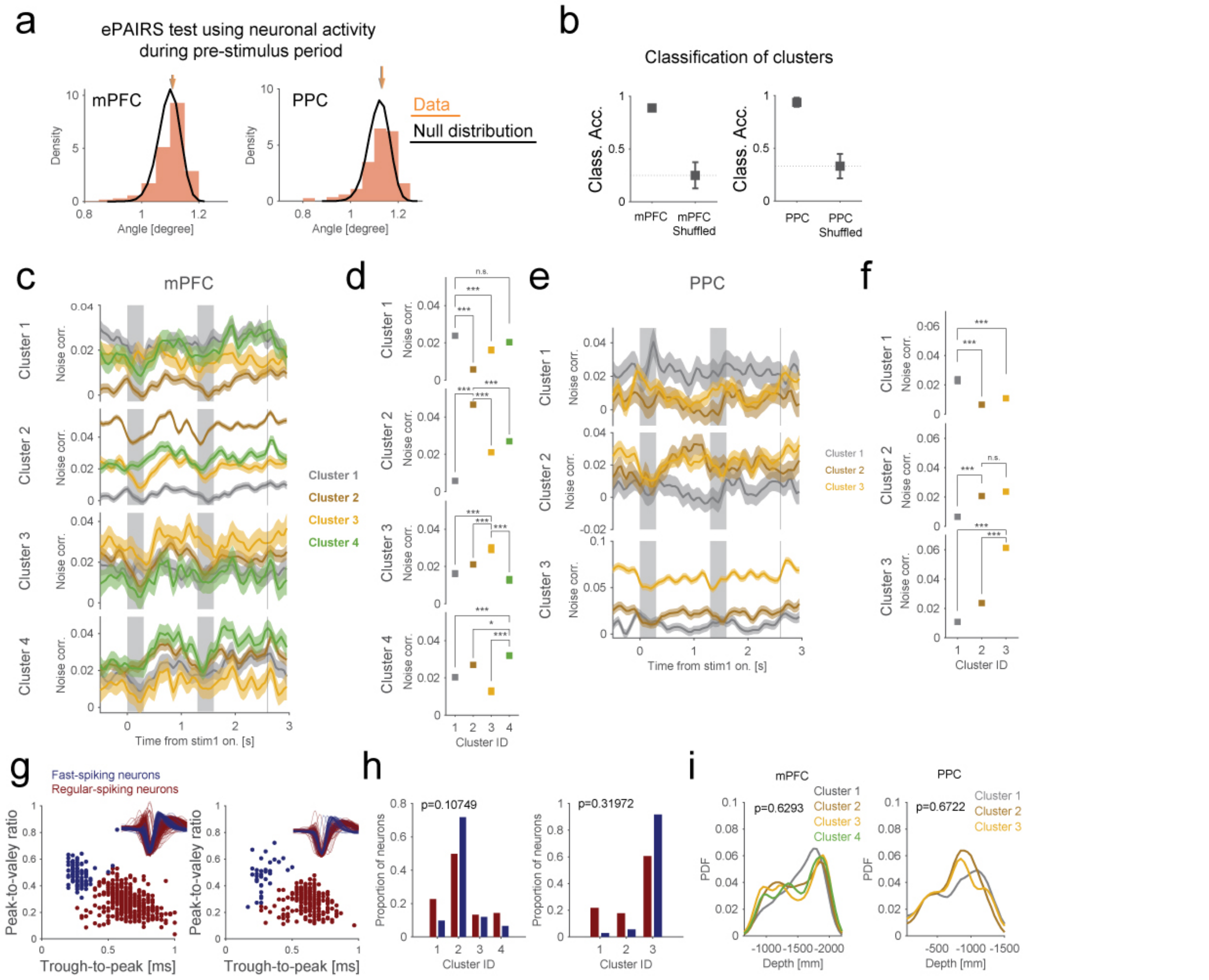

Extended Data Fig.8 |Clustering individual neurons in PPC and classification of cluster ID

(a) The distribution of angles between nearest neighbors calculated using neuronal activity in pre-stimulus periods (-0.5 – 0s from stimulus onset) did not deviate from synthetic null-distribution of response vectors.

(b) Classification accuracy for cluster numbers based on response vectors in the mPFC (left) and PPC (right).

(c) Average noise correlation in each time points in mPFC. The error bars show 95% confidence interval. Each color represents cluster ID.

(d) Average noise correlation across all time points in the mPFC. The error bars show SEM.

(e) Same as **d**, but in PPC.

(f) Same as **e**, but in PPC.

(g) Scatter plot of the trough to (late) peak latency and peak to valley ratio in the mPFC and PPC (Methods). Each color corresponds to putative fast-spiking (FS) inter neurons (blue) and regular-spiking (RS) neurons (red). Inset is the waveforms of FS and RS neurons for each neuron. Spectral clustering was performed to classify neurons into two groups.

(h) Proportion of neurons for each cluster for the FS and RS neurons. There was no difference of distribution between FS and RS neurons.

(i) Depths from the cortical surface for each cluster. There was no significant difference across clusters for both the mPFC and PPC. **i, j.** p-value was computed by one way ANOVA.

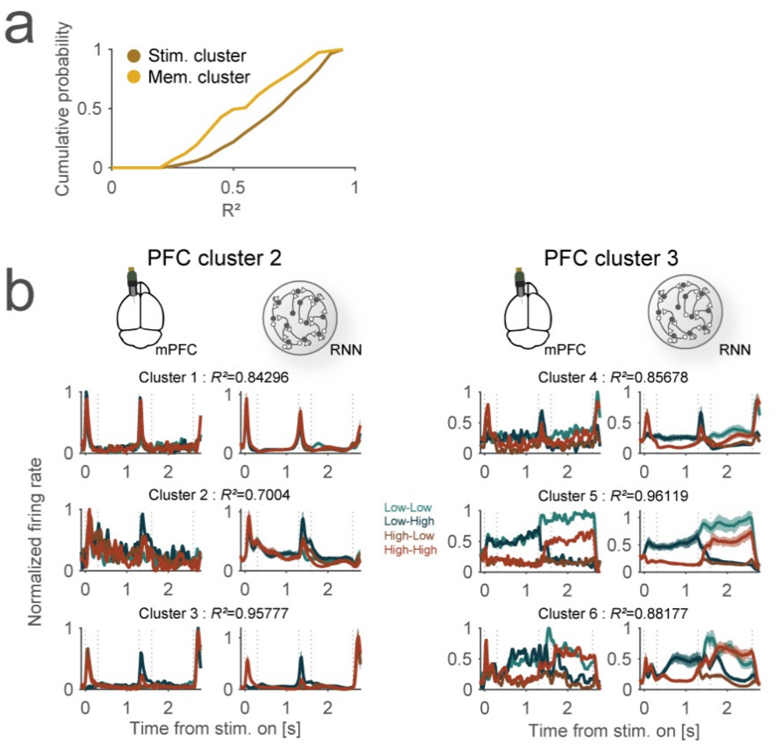

Extended Data Fig.9| Data constrained RNN reliably reproduces neuronal activity in the mPFC
(a) Cumulative probability of R2 values for stimulus processing and memory maintenance clusters computed by PSTHs across four unique trial types between RNN units and mPFC neurons.
(b) Example of RNN unit activities (right) trained to reproduce the PSTH of a recorded mPFC neurons (left) during four unique trial types.

Session number	1	2	3	4	5	6	7	8	9	10	11	12	13	14	15	16	17	18
Mouse ID	1	1	1	2	2	2	3	3	3	3	3	4	4	4	5	6	6	6
# of neurons in mPFC	80	116	61	8	0	66	62	17	5	0	4	48	32	15	32	5	14	21
# of neurons in PPC	32	30	19	0	72	5	7	11	1	0	9	26	29	0	0	0	0	0
# of cluster 1 in mPFC	21	27	11	5	0	15	14	7	2	0	1	4	2	2	6	0	1	2
# of cluster 2 in mPFC	33	55	33	3	0	41	44	9	3	0	2	31	13	6	23	2	7	7
# of cluster 3 in mPFC	11	16	8	0	0	9	2	1	0	0	0	3	2	4	1	2	6	12
# of cluster 4 in mPFC	15	18	9	0	0	1	2	0	0	0	1	10	15	3	2	1	0	0
# of cluster 1 in PPC	6	4	5	0	15	1	3	5	0	0	2	0	4	0	0	0	0	0
# of cluster 2 in PPC	2	8	5	0	19	0	0	1	0	0	1	3	1	0	0	0	0	0
# of cluster 3 in PPC	24	18	9	0	38	4	4	5	1	0	6	23	24	0	0	0	0	0

Supplemental table 1. Number of neurons recorded from each session and cluster ID
